# Synergistic effect of ammonium and potassium on carrot growth, physio-biochemical mechanisms, and water use efficiency under varying irrigation regimes

**DOI:** 10.1038/s41598-025-00690-3

**Published:** 2025-05-09

**Authors:** Ayman M. S. Elshamly, Ahmed D. S. Abaza, Abd El-Zaher M. A. Mustafa, Humaira Rizwana, Javed Iqbal, Shabir Ahmad, Rashid Iqbal, Nazim S. Gruda

**Affiliations:** 1 Ministry of Water Resources and Irrigation, National Water Research Center, Cairo, Egypt; 2 Department of Botany and Microbiology, College of Science, King Saud University, P.O. 2455, Riyadh, 11451 Saudi Arabia; 3 Department of Botany, Bacha Khan University, Charsadda, 24420 Khyber Pakhtunkhwa Pakistan; 4 Department of Plant Sciences, Quaid-i-Azam University Islamabad, Islamabad, 45320 Pakistan; 5 Department of Agronomy, Faculty of Agriculture and Environment, The Islamia University of Bahawalpur, Bahawalpur, 63100 Pakistan; 6 Department of Life Sciences, Western Caspian University, Baku, Azerbaijan; 7 Institute of Plant Sciences and Resource Conservation, Division of Horticultural Sciences, University of Bonn, 53115 Bonn, Germany

**Keywords:** Drought stress, Water use efficiency, Water levels, Physiology, Ammonium hazard, Plant sciences, Environmental sciences

## Abstract

The beneficial effects of ammonium nitrate and potassium humate on carrots are well-documented. However, their impact on physiological and biochemical mechanisms under varying irrigation conditions still needs to be explored. Here, we investigated the effects of soil-applied ammonium nitrate and foliar-applied potassium humate on the physio-chemical characteristics and water use efficiency of carrot plants under three irrigation levels: 100%, 80%, and 60% of crop evapotranspiration (ETc). Carrot plants were treated with two rates of soil ammonium nitrate (200 and 250 kg N ha^− 1^), foliar potassium humate (200 and 400 g 100 L^− 1^), and four combinations of these treatments. Under 80% of ETc, the combined applications of soil ammonium nitrate and foliar potassium humate significantly influenced the leaf contents of chlorophyll a, nitrate, ammonium, catalase, carbohydrate, and soluble sugar patterns, enhancing osmotic regulation under water deficit conditions. Interestingly, when carrots were irrigated by 100% of ETc instead of 80 and 60% and sprayed with 400 g 100 L^− 1^ of potassium humate in combination with 250 kg N ha^− 1^ of ammonium nitrate, water use was decreased by 49.2 and 30.7%, respectively. We attributed that to: a), the observed increments in NH_4_ concentrations in the leaves under 100% ETc which caused negative physiological impacts on chlorophyll, and b) the change in C/N and N/P ratios. This highlights the importance of choosing a suitable irrigation pattern for carrot crops when the potassium humate in combination with ammonium nitrate is adapted. Overall, using foliar potassium humate at a rate of 200 g 100 L^− 1^ with soil ammonium nitrate applications at 250 kg N ha^− 1^ under 80% ETc attained the highest yield and water use efficiency.

**Supplementary Information:**

The online version contains supplementary material available at 10.1038/s41598-025-00690-3.

## Introduction

Nowadays, the biggest challenge for carrot production is the frequency and severity of climate change^1,2^. Decreased soil fertility, rising temperatures, and water deficiency can stress many crops^3,4^. One of the factors reducing carrot yields is water deficiency^5,6^. It has been shown that carrot production decreases by 32.2% for a 20% decrease in water requirements of carrots^7^. However, the comprehensive impacts of soil moisture deficiency on carrots are still being determined^5^. Researchers emphasize that understanding the mechanisms underlying the plant’s response to water levels may be the key to developing strategies to enhance its tolerance and secure food demands in regions affected by water stress in the future^5,8^. Furthermore, it is essential to adopt innovative methods to improve water stress tolerance, especially for water-limited areas^9^. Another challenge is the constantly rising demand for water for purposes and sectors other than agriculture^10^. This challenge will be the main reason for irrigation water shortages in agriculture throughout the coming decades in Egypt^11^. Crop cultivation areas and water demands continually increase while available water resources remain constant^4,12^. Hence, it is essential to help sensitive crops with water deficits, like carrots, adapt to stressful conditions to secure food demand^13^. Adaptation and mitigation practices can be implemented to cope with water stress^14–16^. These include enhanced irrigation efficiency, suitable use of fertilizers^17–19^, and increased emphasis on finding the appropriate combination of both that is more efficient to resist unfavorable growth circumstances^20^.

Globally, carrots are one of the most important vegetable crops. Carrots cultivate more than 10% of arable land annually^21^. However, in Egypt, some problems are associated with increased carrot production, like the shortage of appropriate soil. Therefore, Egyptian farmers employed and promoted coping strategies, such as intensive agriculture practices, crop rotation management, and extension of agricultural lands to desert areas^22^, which are often either sandy or calcareous^20,23,24^. Sandy soils cover over 900 million ha; hence, they occur in many areas worldwide, especially in arid and semi-arid regions^25^. These soils generally have low water-retention capacity, weak soil structure aggregates development, high soil hydraulic conductivity and permeability conductivity, limited nutrient levels, and low organic matter levels^25,26^. Therefore, soil fertility often declines under intensive cultivation and depends on the soil’s organic carbon levels^25^. Despite Baumert et al.^27^ demonstrating that soil organic carbon doesn’t play an essential role in plant nutrient cycling, its role was pronounced in improving soil structure.

To deal with the above mentioned, enhancing the nutrient and water status of sandy soils is essential^28^. This can be achieved by applying organic applications as a source of carbon (e.g., humic acids) and inorganic applications such as ammonium nitrate^29^. In seek of the best agricultural practices, many researchers have sought to mitigate water deficit with the combined use of organic and inorganic components such as humic and calcium nitrate^30^, nitrogen (N) combined with humic acid and iron^31^, humic and urea^32^. Nitrogen fertilizers can be used as a complement to another antitranspirant like potassium humate, and in situations where these combinations are optimized, plant development is often maintained or even improved. Because of the benefits of organic applications were widely utilized to improve growth conditions in sandy soils. A study by Muhammad et al.^33^ has announced that applying humic acids to plants can provide rich nutrient sources and significantly increase plant development and productivity compared to no application. In addition, applying humic acids significantly enhances plant cell membrane permeability and root formation and increases soil water retention capacity, causing improvements in water use efficiency^34,35^. Also, humic applications can have various physiological and biochemical effects on plants, such as increasing the net photosynthetic rate, decreasing the accumulation of reactive oxygen species, enhancing proline synthesis, and increasing the activity of the antioxidant enzyme system^36^. However, others have found that humic applications may cause a non-significant impact on total phenols and total carbohydrates^37^ and proline^38^ or reduce antioxidant activity, resulting in negative impacts on plant growth, especially under higher concentration rates^39^. Consequently, Ampong et al.^40^ found that some study’s recommendations for using humic applications on different crops must be completed, adequate, and consistent. Therefore, it would be preferable for any study that recommends a general benefit of humic applications on soils, crops, or growth conditions to test their actual impacts on the field under specific growth conditions. On the other hand, ammonium nitrate has a significant role in promoting plant growth and development. The principal reasons contributing to the enhancements in plant development and associated physiochemical traits under various growth conditions encompass higher accumulation of carbon and N^41^, increased chlorophyll synthesis and photosynthetic rate^42^, and the generation of total chlorophyll and antioxidants^43^. However, Ma et al.^44^ found that when ammonium nitrate was applied in a different ratio, a significant difference in the examined osmoregulators (soluble protein and free proline contents) was observed, in contrast to the soluble sugars and chlorophyll contents.

Under water deficit circumstances, photosynthesis and transpiration are negatively affected^45^. Each of N and potassium are essential nutrients that significantly regulate physiological processes^46^. Hence, to mitigate the harmful impacts of water deficit conditions, supplying plants with ammonium nitrate and potassium humate is essential. Nonetheless, many plants have differential responses for nutrient combinations throughout their growth circle. Therefore, previous studies indicated that any combinations must be applied with the right proportions to maintain the stoichiometric pattern^47^.

Based on those mentioned above, it can be hypothesized that little data are available about the impact of ammonium nitrate and potassium humate on the physiochemical mechanisms of droughted stressed plants. Plants show many different responses under water deficit conditions. Therefore, considering the above facts, this paper was undertaken to further understand the reaction of carrots, in terms of plant physio-chemical characteristics and water use efficiency, to ammonium nitrate alone and in combination with potassium humate under different irrigation conditions.

## Materials and methods

### The description of the experimental site

**Fig. 1:**
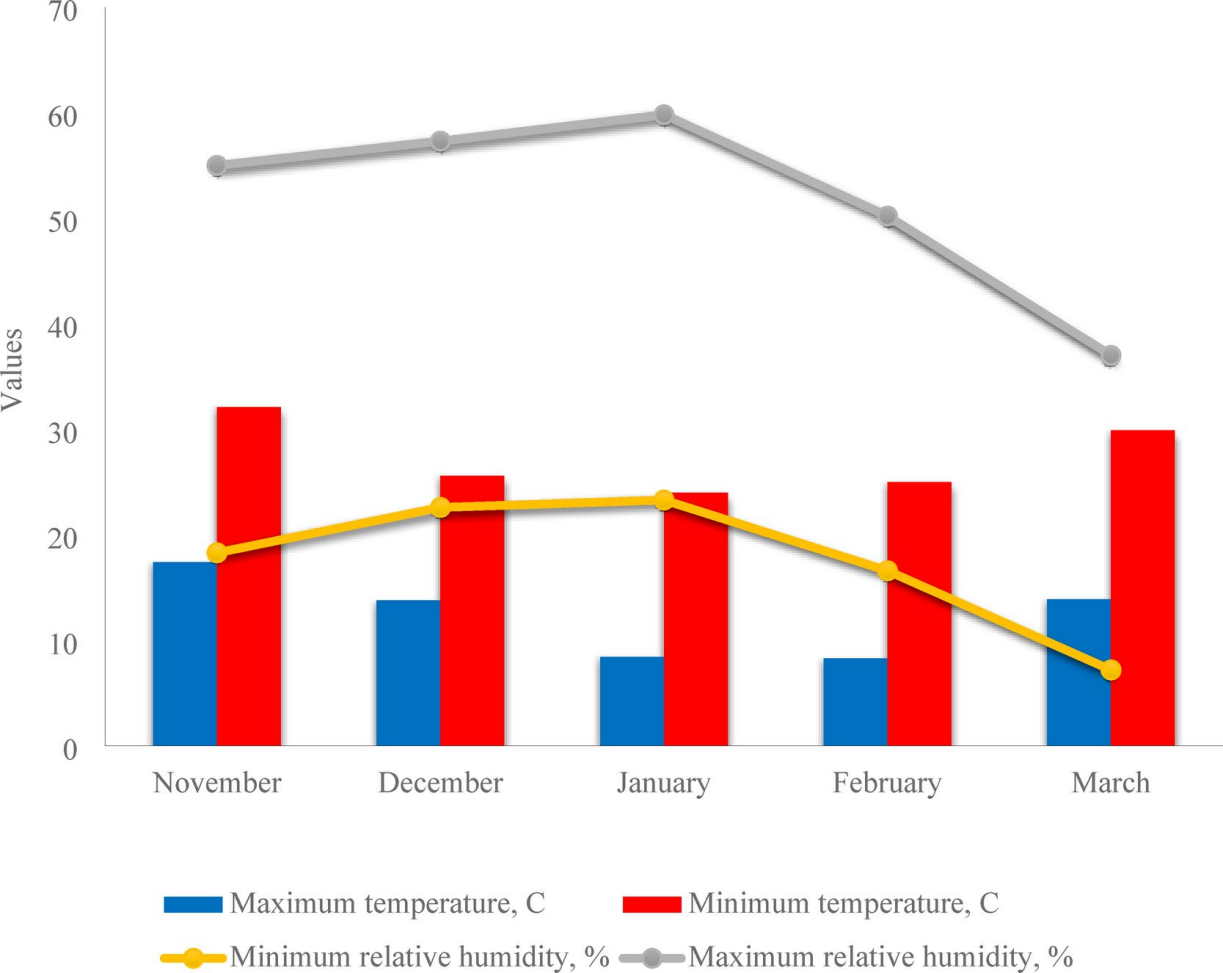
The mean weather data from the experimental site throughout (November to March) during the (2019/2020 and 2020/2021) growing seasons.

This study was conducted at the experimental farm in the South of Egypt at the latitude of 22^o^, 24`0.10` N, longitude of 31o,35`0.41` E, and altitude of 188 m during the seasons of 2019/2020 and 2020/2021. The studied area lies in arid climatic conditions, and the weather data during the cropping seasons are shown in Fig. 1. The soil texture in the experimental station was loamy sand. Soil physical and chemical properties were determined by following Estefan et al.^48^. The soil chemical and physical properties at the experimental field and the qualitative characteristics of irrigation water are shown in Table 1. The farm met its water needs by drawing high-quality groundwater from an on-site deep, with no constraints for the study (Table 2).

**Table 1:** The physicochemical properties of the soil at the experimental site, Egypt, during the growing seasons 2019/2020 and 2020/2021.

Parameter	Unit	Soil depth (cm)		
		0–30	30–60	References
Mechanical analysis				Estefan et al.^48^
Sand	%	86.8	88.7	
Silt	%	3.95	4.15	
Clay	%	9.25	7.15	
Texture	Loamy sand			
Chemical analysis				
pH (1:2.5)		7.92	7.93	
Electrical conductivity (EC)	ds m^− 1^	1.69	0.86	
CaCO_3_	%	1.69	1.56	
Calcium cations (Ca)	mg kg^− 1^	242.5	121.5	
Available Phosphorus (P)	mg kg^− 1^	7.0	6.0	
Available Potassium (K)	mg kg^− 1^	38.0	19.0	
Magnesium cations (Mg)	mg kg^− 1^	109.0	51.0	
Sodium cations (Na)	mg kg^− 1^	704.0	346.0	
Chloride anions (Cl)	mg kg^− 1^	678.0	339.0	
Bicarbonate anions (HCO_3_)	mg kg^− 1^	173.0	90.0	
Sulfate anions (SO_4_)	mg kg^− 1^	237.0	115.0	
Organic matter	%	0.01	0.1	

Each value represents the mean of 3 replications.

**Table 2:** Water chemical properties at the experimental site, Egypt, during the growing seasons 2019/2020 and 2020/2021.

Parameter	Unit	Values	Reference
pH		6.32 ± 0.70	Estefan et al.^48^
TDS	mg L^− 1^	646 ± 3.71	
Electrical conductivity (EC)	ds m^− 1^	1.01 ± 0.05	
HCO_3_	mg L^− 1^	73.2 ± 1.60	
Calcium cations (Ca^+ 2^)	mg L^− 1^	64.1 ± 1.10	
Magnesium cations (Mg^+ 2^)	mg L^− 1^	15.8 ± 0.75	
Sodium cations (Na^+^)	mg L^− 1^	117.3 ± 2.81	
Potassium cations (K^+^)	mg L^− 1^	4.7 ± 0.35	
Chloride anions (Cl^−^)	mg L^− 1^	113.5 ± 3.35	
Sulfate anions (SO_4_ ^− 2^)	mg L^− 1^	240.2 ± 2.10	
SAR		3.40 ± 0.10	

Each value represents the mean of replications ± standard errors. Abbreviations: TDS: total dissolved solids; SAR: the sodium adsorption ratio.

### The experimental details

This field experiment was performed under different irrigation levels to examine the efficiency of two rates of ammonium nitrate as a soil application (SAN) and two rates of foliar potassium humate (FKH) on carrot plants. The experiment layout was a split plot based on a randomized complete block design with three replications. Irrigation levels were determined at three levels as the main plots {100%, 80%, and 60% of carrot crop evapotranspiration (ETc)}. Concurrently, in the subplots carrot plants were treated with: T1 (tap water applications) (as control), T2 (SAN = 200 kg N ha^− 1^), T3 (SAN = 250 kg N ha^− 1^), T4 (FKH = 200 g 100 L^− 1^ water), T5 (FKH = 400 g 100 L^− 1^ water), T6 (SAN + FKH = 200 kg N ha^− 1^+ FKH = 200 g 100 L^− 1^ water), T7 (SAN + FKH = 200 kg N ha^− 1^+ FKH = 400 g 100 L^− 1^ water), T8 (SAN + FKH = 250 kg N ha^− 1^+ FKH = 200 g 100 L^− 1^ water), and T9 (SAN + FKH = 250 kg N ha^− 1^+ FKH = 400 g 100 L^− 1^ water). Irrigation in the current study was implemented through a set sprinkler irrigation system using a metal impact sprinkler 3/4” male (Rain Bird Sprinkler, U.S.A) with a discharge of 1.2–1.4 m^3^ h^− 1^ at 3 bars. Each plot had a manometer valve to control the operating pressure at 3 bar. Beside a flow emitter and valve to control the mounts of the targeted irrigation water level. Moreover, to avoid the potential impacts of water infiltration on the adjacent plots, a buffer zone of nine meters was meticulously maintained between the experimental treatments. The experimental net space was (9.0 m long × 9.0 m wide). Additionally, the experimental work involved 81 plots {3 irrigation levels × 9 fertilization treatments × 3 replicates}. Carrot seeds were purchased from Takii Seeds Co. This cultivar is recommended as a high-yielding commercial cultivar. Moreover, this cultivar and the implemented methods in the current study complied with international, national, and institutional guidelines and legislation: the carrot seeds (*Daucus carota* L.), cv. Kuroda Max was sown in 1st week of November during the first and second seasons of 2019/2020 and 2020/2021, respectively. The carrot plant density in the current study was established at a rate of approximately 0.8 million plants per hectare. The roots were harvested on March 10, 2020, and March 15, 2021. The experimental area was divided into two parts to examine the effects of the fertilizer treatments: one part was used in the first year, and the other was used in the second year. Commercially available fertilizers were applied, where phosphorus in calcium superphosphate (16% P_2_O_5_) form was applied at 40 kg P_2_O_5_ ha ^− 1^ in two equaled portions during seedbed preparation and after 30 days of planting date. Both of soil amounts of N (200 and 250 kg N ha^− 1^) in the form of ammonium nitrate (33.5% N) and 125 kg K_2_O ha ^− 1^ of potassium sulfate (48% K_2_O) were divided into equal portions, and applied once 30 days after emergence in the stem elongation stage (20–39, BBCH code) and after 60 days of emergence in the flowering stage (40–89, BBCH code). The FKH product was purchased from Farma Egypt Co, and it had 80.0% humic substances involving 68.0% humic acid, 5% fulvic acid, pH 9.5, and 10.0% potassium. Both FKH doses (200 and 400 g 100 L^− 1^ water) were sprayed and applied twice in the same concentrations after 60 and 75 days from the emergence in the flowering growth stage (40–89, BBCH code). Throughout the growing seasons, the district’s commercial carrot production regularly conducted all relevant agricultural practices, including plant protection practices against disease, weeds, and pests.

### The irrigation calculation procedures

Determining the irrigation water requirement involved downloading the actual weather data obtained from the Toshka agrometeorological station and calculating the crop evapotranspiration of carrots to establish the exact amount for subsequent irrigation events for each treatment. The quantity of irrigation water for each treatment was calculated using the following equations:

### Reference evapotranspiration

The daily reference evapotranspiration (ETo) was calculated by entering weather data in the CROPWAT package, version 8.0. Then, ETo was determined by Penman-Monteith equation as indicated by Allen et al.^49^:

$$\:\text{E}\text{T}\text{o}=\frac{0.408{\Delta\:}\:\left(\text{R}\text{n}-\text{G}\right)+{\upgamma\:}\:\frac{900}{T+273}\text{U}2\:(\text{e}\text{s}-\text{e}\text{a})\:}{{\Delta\:}+{\upgamma\:}\:(1+0.34\text{U}2)}$$

Where:

ETo = Reference evapotranspiration (mm day^− 1^), Rn = Net radiation (MJm^− 2^d^− 1^), G = Soil heat flux (MJm^− 2^d^− 1^), Δ = Slope vapor pressure and temperature curve (kPa ^o^C^−1^), γ = Psychrometric constant (kPa °C^− 1^), U2 = Wind speed at 2 m height (ms^− 1^), es-ea = Vapor pressure deficit (kPa), T = Mean daily air temperature at 2 m height (°C).

### Crop evapotranspiration

The crop evapotranspiration of carrots (ETc) was calculated based on Allen et al.^46^ as the following equation:

$${\text{ETc}} = \left( {{\text{ETo }} \times {\text{ Kc}}} \right)$$

Were ETc = Crop evapotranspiration (mm), ETo = Reference evapotranspiration (mm), Kc = Crop coefficient.

Before the experiment started, soil moisture parameters were determined, and a reduction in soil moisture was allowed to 60% of the available water, which was the critical limit on carrot development based on a previous study^7^. Consequently, the irrigation water intervals were carried out every two days. Moreover, in the two winter growing seasons of 2019/2020 and 2020/2021, the applied irrigation water for carrot plants was determined using the reference plots where 100% of ETc was maintained. Subsequently, the stressful irrigation treatments were calculated by applying pre-determined irrigation levels. Specifically, stressful irrigation levels were established at 80% and 60% of the ETc. Accordingly, the average two-season quantities of applied irrigation water were 4241, 3393, and 2545 m^3^ ha^− 1^ for 100, 80, and 60%, respectively. Irrigation regime levels were started in the stem elongation stage (20–39, BBCH code) after 45 days of emergence, namely when the carrot plants had reached the end of the true leaf growth stage, characterized by the complete formatting and opening of the third genuine leaf^50^.

### Biochemical measurements

The youngest fully expanded carrot leaves (fourth or fifth leaf from the top) were chosen at the flowering growth stage (40–89, BBCH code) after 65 days of emergence (DAE) and before the physiological maturity stage (120 DAE).

### Estimation of chlorophyll a, b, and total carotenoids

At 65 and 120 DAE, carrot chlorophyll a and b contents in the leaves were measured via spectrophotometer based on^51^. 100 mg of fresh carrot leaves were collected, crushed, pulverized, and dissolved in 10 ml acetone 80%, then left in the dark for 48 h to extract the chlorophyll. For five minutes, the obtained extract was centrifuged at 3000 rpm. After that, chlorophyll a, chlorophyll b, and total carotenoids were measured using a spectrophotometer at a wavelength of 645, 663, and 470 nm, respectively.

### Estimation of soluble sugars and carbohydrate

Carrot leaf samples were dried and crushed into a fine powder. Carbohydrate extraction and estimation in the leaves were based on the Anthrone method^52^. The 0.5 g of the powdered material was boiled with 5 ml of 80% ethanol and incubated for 30 min at 80 °C for soluble sugars extraction. The mixture was decanted after centrifugation for 10 min, followed by the extraction. The residual was resuspended in 80% ethanol. Then, the same procedure was repeated twice. After that, the supernatant was thoroughly mixed with anthrone (8.6 mM anthrone in 80% v/v H_2_SO_4_) as a reagent at 620 nm. Finally, the absorbance was quantified using the same method as soluble sugars.

### Estimation of proline

Proline concentration was determined as described by Bates et al.^53^. The 0.5 g of fresh leaf samples were extracted from carrot leaf tissues by 0.6 ml of 80% hot ethanol. After that, the obtained extract was mixed with 10 ml of 3% sulfosalicylic acid, and the solution was homogenized in a water bath at 99 °C. After centrifugation at 23 000 × g for 10 min, 2 ml of the supernatant was taken and added to 2 ml of glacial acetic acid and 3 ml of 2.5% ninhydrin reagent. In boiling water, the reaction mixture was incubated for one hour. After adding 4 ml of toluene, the absorbance was measured using a spectrophotometer at 520 nm, and the pellet was re-extracted three times. The mean value of proline contents was expressed as µ mol g^− 1^ FW.

### Estimation of ammonium (NH_4_)

To measure NH_4_ in carrot leaves, 0.5 g was taken, homogenized under liquid nitrogen with 6 ml of 10 mM formic acid, and left for five minutes. After repeating centrifugation at 4 °C and 12,000 rpm × g for 10 min 3 times, the supernatant previously described by Shi et al.^54^ was diluted with 2.5 ml o-phthalaldehyde (OPA) solution. Finally, by spectrophotometer, the absorbance of the sample was measured at 410 nm. To calculate NH4 + concentrations in plant leaves, the standard formula of Li et al.^55^ was used.

### Estimation of Nitrate (NO_3_)

Based on Zhao and Wang’s^56^ method, frozen (0.1 g) leaf samples were added to 1 ml deionized water and boiled at 100 °C for 20 min at least. After centrifugation at 15,871 x g for 10 min, 0.1 ml of the supernatant was transferred into a new 12-ml tube, and then 0.1 ml of deionized water was used as a control. 0.4 ml salicylic acid and sulphuric acid (H_2_SO_4_) were added in each tube. Then, the sample was vortexed well and incubated for 20 min at room temperature. After that, in each tube, 9.5 ml of 8% (w/v) NaOH solution was added and cooled down for 20–30 min at room temperature. Then, the nitrate contents of each sample, with the control for reference, were measured using a spectrophotometer at 410 nm.

### Estimation of hydrogen peroxide (H_2_O_**2**_)

For the H_2_O_2_ assay, frozen leaf materials (0.5 g) were homogenized in KH_2_PO_4_-KOH buffer (pH 7.8). Then, using titanium chloride (TiCl_2_) and a spectrophotometer, the optical density was measured at 410 nm. The same procedure was repeated on the standards, which contained no plant material in the range of 0.75 to 5.35 mM H_2_O_2_ [Estefan et al.^48^.

### Estimation of antioxidant enzymes

The antioxidant activity determinations were estimated based on the methods of Azevedo Neto et al.^57^ and Ahsan et al.^58^. To confirm the results, three replicates for each treatment were used. The carrot leaves in each experimental unit were randomly selected from ten different plants. And antioxidants results were expressed in µ mol g^− 1^ FW.

### The activity of catalase (CAT)

A spectrophotometer was used to determine CAT activity at a wavelength of 240 nm. The reaction mixture consisted of 3 ml of 50 mM potassium phosphate buffer added to 1 g of fresh carrot leaves, then mixed with 30% H_2_O_2_ (10 µl) and 50 µl of protein extract. For 5 min, the CAT activity was monitored based on Aebi’s^59^, and the readings were recorded at 20-s intervals. And by monitoring H_2_O_2_ degradation in the absorbance range of one minute at 240 nm via spectrophotometer.

### The activity of superoxide dismutase (SOD)

To estimate the activity of SOD enzyme in carrot leaves, 1 g of fresh leaves was crushed into fine powder and homogenized for five minutes with a reaction mixture consisting of (K-phosphate buffer pH 6.8 + 0.1 mm EDTA). After filtration, the mixture was centrifuged at 16,000 × g for fifteen minutes, then 50 µl of the enzymatic extract was added to 75 μm nitro blue tetrazolium chloride (NBT) + 13 mm l-methionine + 100 μm EDTA + 2 μm riboflavin in a 50 mm potassium phosphate buffer pH 7.8. Then SOD enzyme activity was measured by a spectrophotometer at a wavelength of 560 nm.

### The activity of peroxidase (POD)

To determine POD enzyme activity, a reaction mixture consisted of 50 ml + 3 ml phosphate buffer (0.1 M, pH 7.0) + 30 ml 2% H_2_O_2_ + 50 ml guaiacol (20 mM). After adding 2.0 ml of 20% chloroacetic acid, an increase in absorbance at 470 nm was recorded.

### The activity of ascorbate peroxidase (APX)

Regarding the evaluation of APX activity, a reaction mixture was composed of 1500 µl of 50 mM potassium phosphate buffer (pH 6.0) + 600 µl of 0.1 mM EDTA + 10 µl of 30% H_2_O_2_ + 400 µl of 0.5 mM ascorbic acid + 50 µl of protein extract. The reaction was initiated by monitoring H_2_O_2_ degradation every 20 s for two minutes at 290 nm using a spectrophotometer^60^.

### Dry matter determination

Based on the method described by Shipley & Vu^61^, five grams of fresh carrot samples (leaves - roots) were washed in deionized water, oven-dried to a constant weight at 70 °C at least 48 h, and then weighed again (dry weight). After that, total dry matter values were obtained by summing the relevant carrot organs.

### Yield

At the harvest, one m^− 2^ of root samples were taken from each sub-plot, weighted then carrot yield was converted to kg ha^− 1^, and each sampling comprised 3 replicates.

### Water use efficiency (WUE) calculation

The WUE was determined according to^62^

$${\text{WUE}} = {\text{~}}\left( {\frac{{\text{Y}}}{{{\text{ETc~}}}}} \right)$$

where WUE = Water use efficiency (kg m^− 3^), Y = Root yield (kg ha^− 1^) and ETc = Crop evapotranspiration.

### The statistical analysis

Combined data of the two growing seasons were performed to analyze the individual and interactive impacts of the differences among the examined application and irrigation levels on the examined variables through split plot design (two-way ANOVA) in the statistical package Costat version 6.303. The significant differences were maintained at 0.05 to compare treatment means with the post hoc Tukey test, as per Casella et al.^63^. Lowercase letters above error bars indicate statistically significant differences (*p* < 0.05).

## Results

### The impacts of SAN and FKH under various irrigation levels

#### Chlorophyll a, b, and total carotenoids

**Fig. 2:**
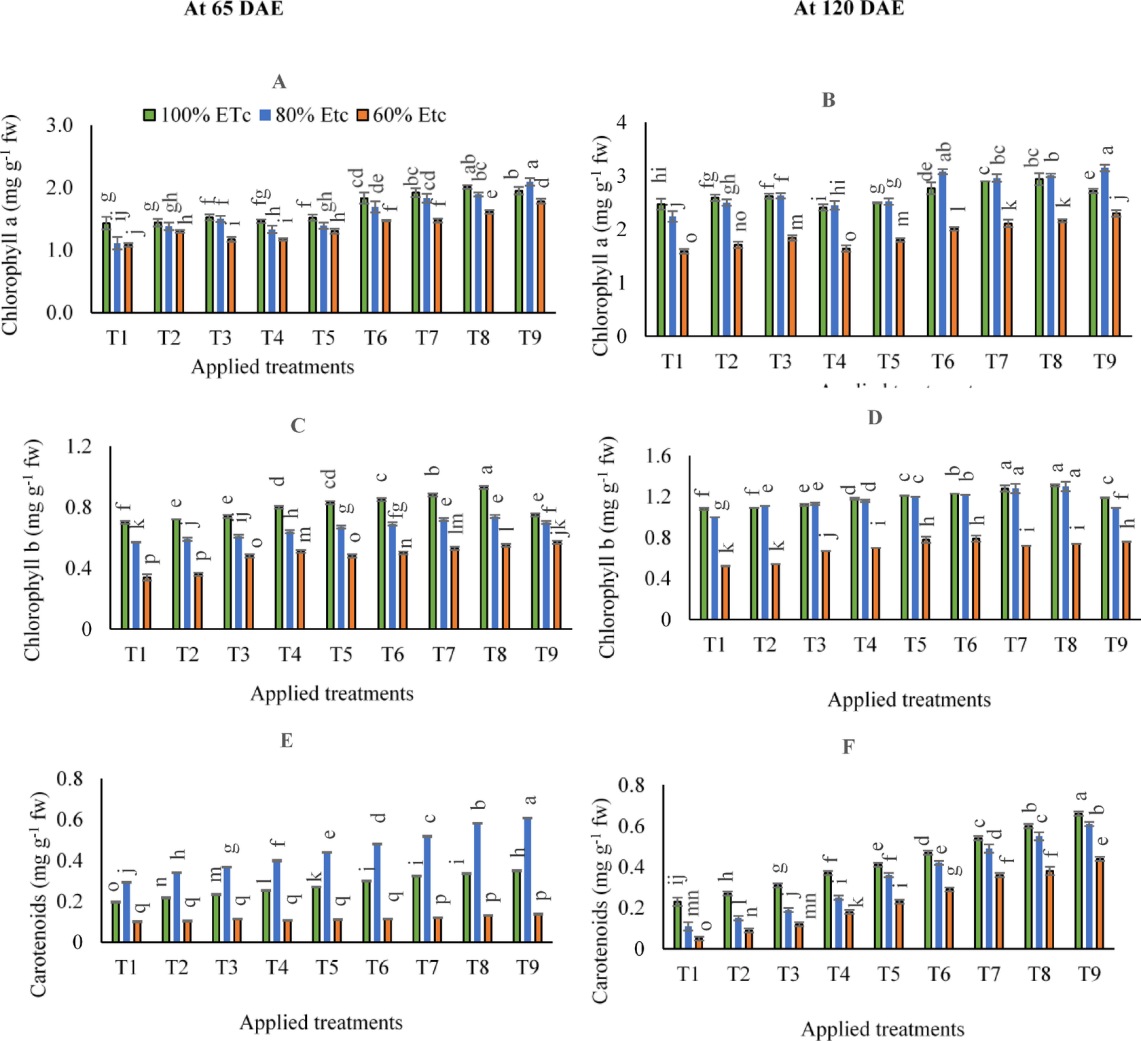
The mean interactive impacts of irrigation water levels and different applications and rates of [soil ammonium nitrate (SAN) and foliar potassium humate (FKH)] as individual or combined applications during the growing seasons (2019/2020 and 2020/2021) on (**A**) Chlorophyll a- 65 DAE, (**B**) Chlorophyll a- 120 DAE, (**C**) Chlorophyll b- 65 DAE, (**D**) Chlorophyll b- 120 DAE, (**E**) Carotenoids − 65 DAE, and (**F**) Carotenoids − 120 DAE. Error bars indicate standard errors from the mean. Bars with different letters are statistically significant at *p* ≤ 0.05. Abbreviations: T1 (tap water applications) (as control), T2 (SAN = 200 kg N ha^− 1^), T3 (SAN = 250 kg N ha^− 1^), T4 (FKH = 200 g 100 L^− 1^ water), T5 (FKH = 400 g 100 L^− 1^ water), T6 (SAN + FKH = 200 kg N ha^− 1^+ FKH = 200 g 100 L^− 1^ water), T7 (SAN + FKH = 200 kg N ha^− 1^+ FKH = 400 g 100 L^− 1^ water), T8 (SAN + FKH = 250 kg N ha^− 1^+ FKH = 200 g 100 L^− 1^ water), and T9 (SAN + FKH = 250 kg N ha^− 1^+ FKH = 400 g 100 L^− 1^ water).

Based on the results of variance analysis (Supplementary Table S1), the impacts of various irrigation levels and fertilized treatments (T_1_:T_9_) on chlorophyll a, b, and total carotenoid contents at 65 DAE and 120 DAE were significant (*p* < 0.05). The results in (supplementary Table S2**)** show the individual effects of adopting ammonium nitrate and potassium humate under different irrigation levels on (chlorophyll a, chlorophyll b, and carotenoids) in carrot leaves. While Fig. 2 shows the interactive impacts on (chlorophyll a, chlorophyll b, and carotenoids) in carrot leaves. As can be seen in (Fig. 2A, B, C, and D), in contrast to 100% ETc; irrigated carrot plants with 80 or 60% ETc under control treatment at 65 and 120 DAE showed significant reductions in average chlorophyll a and b contents. While executing 80% ETc irrigation level was pronounced in terms of yielding higher carotenoid contents at 65 DAE compared to 100 or 60% ETc irrigation levels (Fig. 2E). However, at 120 DAE, carotenoid contents were significantly increased by adopting 100% ETc and decreased by adopting 80 or 60% ETc irrigation levels (Fig. 2F).

Data in Fig. 2C showed that by adopting 100% of ETc at 65 DAE, the highest chlorophyll b contents were observed by applying combined applications of (250 kg N ha^− 1^+ 200 g FKH 100 L^− 1^ water- T8). Likewise, the maximum chlorophyll b contents after 120 DAE were obtained by applying combined applications of (250 kg N ha^− 1^+ 200 g FKH 100 L^− 1^ water- T8) under 100 and 80% of ETc, which were significantly equaled by applying combined applications of (200 kg N ha^− 1^+ 400 g FKH 100 L^− 1^ water- T7).

On the other hand, in contrast to the short period, when it comes to maximizing carotenoid contents in the long term (at 120 DAE), the higher application rates and irrigation levels remain preferable. According to the results in (Fig. 2A), executing 100% of ETc irrigation level and applying the combined applications of (250 kg N ha^− 1^+ 400 g FKH 100 L^− 1^ water- T9) were statistically yielding greater carotenoid contents.

### H_2_O_2_, NO_3_, and NH_4_

**Fig. 3:**
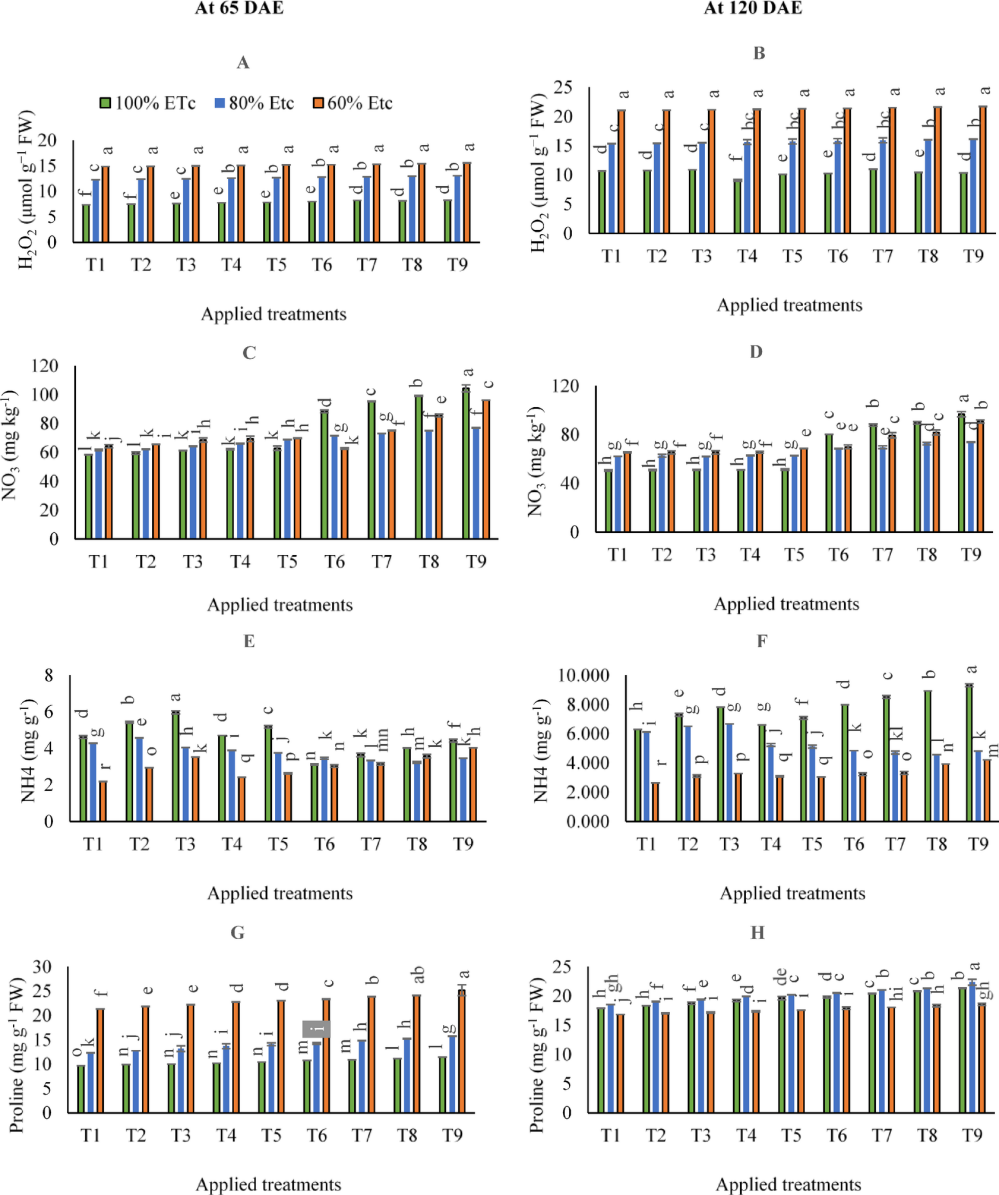
The mean interactive impacts of irrigation water levels and different applications and rates of [soil ammonium nitrate (SAN) and foliar potassium humate (FKH)] as individual or combined applications during the growing seasons (2019/2020 and 2020/2021) on (**A**) H_2_O_2_- 65 DAE, (**B**) H_2_O_2_- 120 DAE, (**C**) NO_3_- 65 DAE, (**D**) NO_3_ − 120 DAE, (**E**) NH_4_ − 65 DAE, (**F**) NH_4_ − 120 DAE, (**G**) Proline − 65 DAE, and (**H**) Proline − 120 DAE. Error bars indicate standard errors from the mean. Bars with different letters are statistically significant at *p* ≤ 0.05. Abbreviations: T1 (tap water applications) (as control), T2 (SAN = 200 kg N ha^− 1^), T3 (SAN = 250 kg N ha^− 1^), T4 (FKH = 200 g 100 L^− 1^ water), T5 (FKH = 400 g 100 L^− 1^ water), T6 (SAN + FKH = 200 kg N ha^− 1^+ FKH = 200 g 100 L^− 1^ water), T7 (SAN + FKH = 200 kg N ha^− 1^+ FKH = 400 g 100 L^− 1^ water), T8 (SAN + FKH = 250 kg N ha^− 1^+ FKH = 200 g 100 L^− 1^ water), and T9 (SAN + FKH = 250 kg N ha^− 1^+ FKH = 400 g 100 L^− 1^ water).

According to the statistical analysis, the impacts of various irrigation levels and examined applications on H_2_O_2_, NO_3_, and NH_4_ contents at 65 and 120 DAE were significant (*p* < 0.05). By comparing irrigation water levels in the control treatments in (Fig. 3), adopting 80 and 60% of ETc resulted in significant increases in H_2_O_2_ and NO_3_ compared to 100% of ETc at 65 DAE and 120 DAE, as can be seen in (Fig. 3A, B, C, and D); in contrast to NH_4_ (Fig. 3E and F).

By comparing the individual applications of SAN and FKH at 65 DAE, applying both individual applications of FKH under 100 and 80% of ETc and the higher rate of SAN under 100% of ETc; led to significant increases in H_2_O_2_ contents compared to the same irrigation levels in the control treatment. At the same time, there was no significant effect in the remaining treatments. At 120 DAE, the obtained results in (supplementary Table S3**)** showed that any individual applications of SAN and FKH under 80 and 60% of ETc, didn’t attain significant impacts on H_2_O_2_ contents compared to the same irrigation levels in the control treatment. However, when adopting 100% of ETc and applying the individual applications of FKH at a rate of (200 g 100 L^− 1^ water- T4), H_2_O_2_ content attained a lower significant value. On the other hand, collectively adopting (60% of ETc) with or without applying any individual and combined applications of SAN and FKH achieved the maximum increase of H_2_O_2_ contents at 65 and 120 DAE.

Generally, the results in (Fig. 3C and D) showed that applying the combined applications of (250 kg N ha^− 1^+ 400 g FKH 100 L^− 1^ water- T9 under 100% of ETc) after 65 and 120 DAE resulted in the highest NO_3_ concentration compared to the other treatments.

On the other hand, it was shown that the maximum significant NH_4_ accumulations could be observed by applying the individual applications of (SAN at the rate of 250 kg N – T3 under 100% of ETc) and applying the combined applications of (250 kg N ha^− 1^+ 400 g FKH 100 L^− 1^ water- T9 under 100% of ETc) after 65 and 120 DAE, respectively (Fig. 3E and F).

### Proline and antioxidant enzymes

The obtained results in (supplementary Tables S3 & S4**)** and (Fig. 3G and H**)** indicated that adopting stressful irrigation levels 80 and 60% of ETc led to increased proline content after 65 DAE. Interestingly, after 120 DAE, irrigated carrot plants with 100 and 80% ETc irrigation levels showed the highest average proline contents. After 65 DAE, treatment carrot plants with the individual applications of FKH showed higher proline contents under 80 and 60% of ETc irrigation levels. However, applying any individual application of SAN and FKH under 100% of ETc irrigation level improved proline contents better than control 100% of ETc. Overall, after 65 DAE, the highest proline content was obtained by adopting 60% of ETc and applying combined applications of combined applications of (250 kg N ha^− 1^+ 400 g FKH 100 L^− 1^ water- T9). However, that significantly equaled adopting the same irrigation level and applying the combined application of (250 kg N ha^− 1^+ 200 g FKH 100 L^− 1^ water- T8). After 120 DAE, the highest proline content was seen using combined applications of (250 kg N ha^− 1^+ 400 g FKH 100 L^− 1^ water- T9) and adopting 80% of ETc irrigation level.

**Fig. 4:**
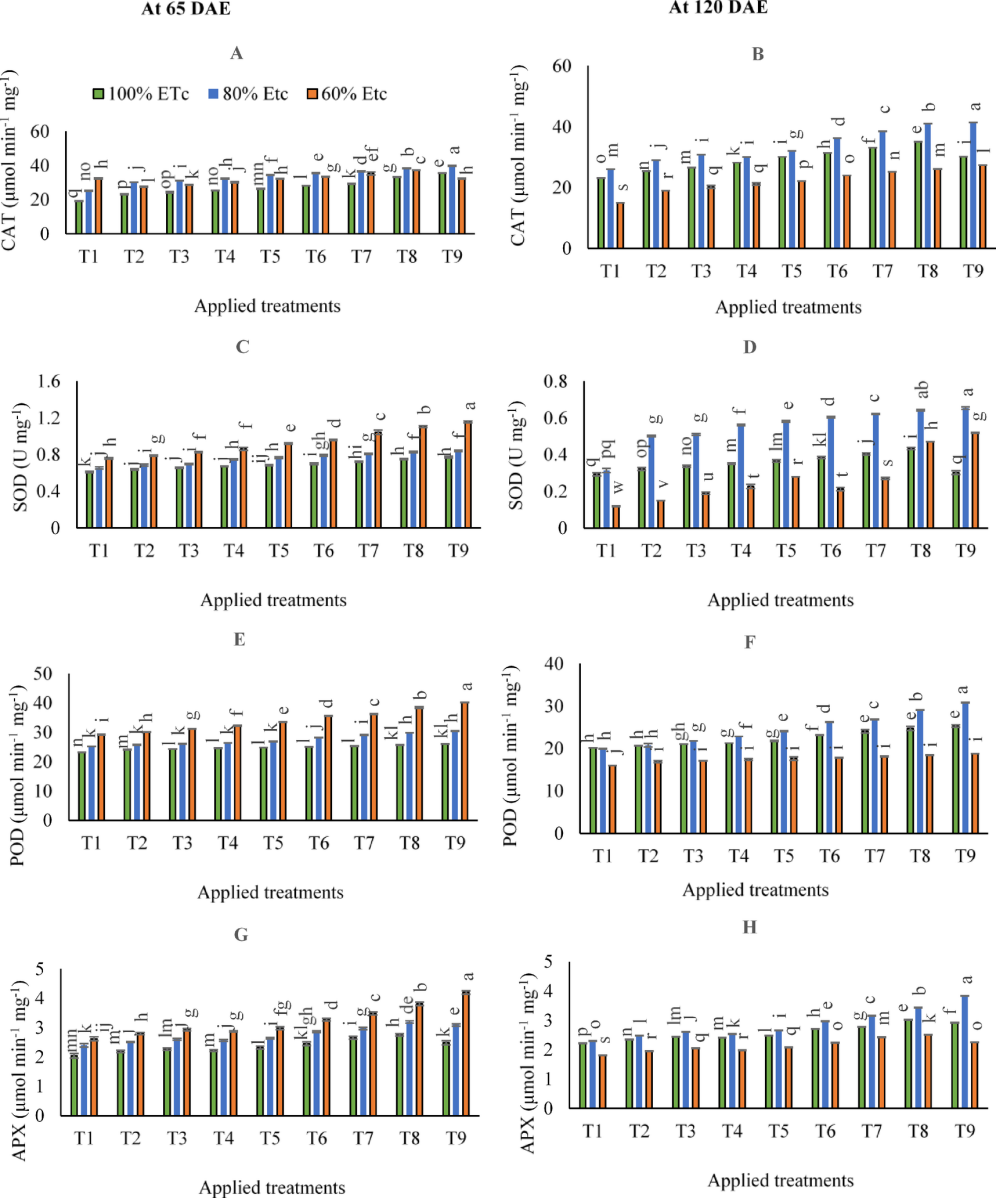
The mean interactive impacts of irrigation water levels and different applications and rates of [soil ammonium nitrate (SAN) and foliar potassium humate (FKH)] as individual or combined applications during the growing seasons (2019/2020 and 2020/2021) on antioxidant enzymes (**A**) CAT- 65 DAE, (**B**) CAT- 120 DAE, (**C**) SOD- 65 DAE, (**D**) SOD- 120 DAE, (**E**) POD − 65 DAE, (**F**) POD- 120 DAE, (**G**) APX − 65 DAE, and (**H**) APX- 120 DAE. Error bars indicate standard errors from the mean. Bars with different letters are statistically significant at *p* ≤ 0.05. Abbreviations: T1 (tap water applications) (as control), T2 (SAN = 200 kg N ha^− 1^), T3 (SAN = 250 kg N ha^− 1^), T4 (FKH = 200 g 100 L^− 1^ water), T5 (FKH = 400 g 100 L^− 1^ water), T6 (SAN + FKH = 200 kg N ha^− 1^+ FKH = 200 g 100 L^− 1^ water), T7 (SAN + FKH = 200 kg N ha^− 1^+ FKH = 400 g 100 L^− 1^ water), T8 (SAN + FKH = 250 kg N ha^− 1^+ FKH = 200 g 100 L^− 1^ water), and T9 (SAN + FKH = 250 kg N ha^− 1^+ FKH = 400 g 100 L^− 1^ water).

Concerning the antioxidant enzymes, the results in (Fig. 4A, B, C, D, E, F, G, and H) showed that executing control 60% of ETc after 65 DAE was statistically significant in terms of yielding higher CAT, SOD, POD, and APX contents, compared to control treatments for 100 and 80% of ETc. Conversely, after 120 DAE, it was shown that adopting 100 or 80% ETc irrigation levels could achieve higher antioxidant accumulations.

On the other hand, the findings indicated that by adopting 80% of ETc, the maximum CAT contents were observed by applying combined applications of (250 kg N ha^− 1^+ 400 g FKH 100 L^− 1^ water- T9) after 65 and 120 DAE. On the other side, when it comes to maximizing SOD contents, the maximum contents were observed by applying combined applications of (250 kg N ha^− 1^+ 400 g FKH 100 L^− 1^ water- T9) under 60% of ETc after 65 DAE. While after 120 DAE, applying combined applications of (250 kg N ha^− 1^+ 400 g FKH 100 L^− 1^ water- T9) under 80% of ETc was pronounced in terms of yielding greater SOD contents, although that significantly equaled the adoption same irrigation level and applying the combined application of (250 kg N ha^− 1^+ 200 g FKH 100 L^− 1^ water- T8).

A similar finding was observed for POD and APX contents. In contrast, the results indicated that the maximum contents were observed by applying combined applications of (250 kg N ha^− 1^+ 400 g FKH 100 L^− 1^ water- T9) under 60% of ETc irrigation level after 65 DAE. And by applying combined applications of (250 kg N ha^− 1^+ 400 g FKH 100 L^− 1^ water- T9) under 80% of ETc after 120 DAE.

### Dry matter, carbohydrate, and soluble sugars

**Fig. 5:**
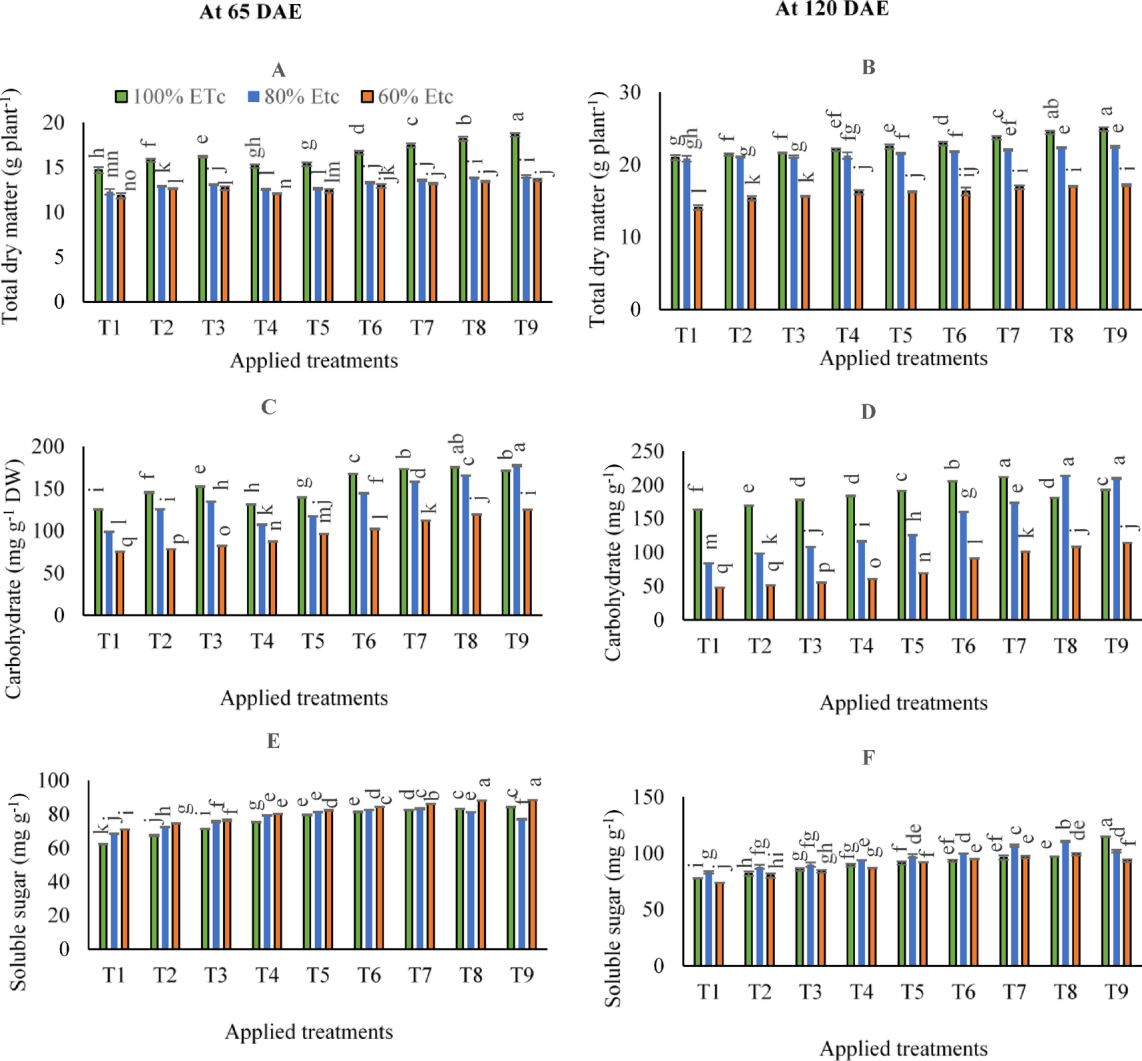
The mean interactive impacts of irrigation water levels and different applications and rates of [soil ammonium nitrate (SAN) and foliar potassium humate (FKH)] as individual or combined applications during the growing seasons (2019/2020 and 2020/2021) on (**A**) Dry matter − 65 DAE, (**B**) Dry matter − 120 DAE, (**C**) Carbohydrate − 65 DAE, (**D**) Carbohydrate − 120 DAE, (**E**) Soluble sugars − 65 DAE, and (**F**) Soluble sugars − 120 DAE. Error bars indicate standard errors from the mean. Bars with different letters are statistically significant at *p* ≤ 0.05. Abbreviations: T1 (tap water applications) (as control), T2 (SAN = 200 kg N ha^− 1^), T3 (SAN = 250 kg N ha^− 1^), T4 (FKH = 200 g 100 L^− 1^ water), T5 (FKH = 400 g 100 L^− 1^ water), T6 (SAN + FKH = 200 kg N ha^− 1^+ FKH = 200 g 100 L^− 1^ water), T7 (SAN + FKH = 200 kg N ha^− 1^+ FKH = 400 g 100 L^− 1^ water), T8 (SAN + FKH = 250 kg N ha^− 1^+ FKH = 200 g 100 L^− 1^ water), and T9 (SAN + FKH = 250 kg N ha^− 1^+ FKH = 400 g 100 L^− 1^ water).

To compare the irrigation levels differences in dry matter contents after 65 and 120 DAE, the results in (supplementary Table S5) and (Fig. 5A and B) showed that dry matter contents were decreased when adopting 80 and 60% of ETc compared to control 100% of ETc after 65 DAE. After that, in contrast to 60% of ETc, adopting 80% of ETc significantly equaled and attained the same improvements in dry matter contents.

Collectively, applying the combined applications of (250 kg N ha^− 1^+ 400 g FKH 100 L^− 1^ water- T9 under 100% of ETc) after 65 DAE resulted in the highest dry matter content compared to the other treatments. After 120 DAE, the maximum dry matter contents were obtained by applying combined applications of (250 kg N ha^− 1^+ 400 g FKH 100 L^− 1^ water- T9 under 100% of ETc), although that was significantly equaled by applying combined applications of (250 kg N ha^− 1^+ 200 g FKH 100 L^− 1^ water- T8 under 100% of ETc).

Based on the results of variance analysis (Supplementary Table S1) and (supplementary Table S5), the individual and interaction effects of examined irrigation levels and various applications significantly affected the carbohydrate and soluble sugar concentrations.

As can be seen in (Fig. 5C), it was shown that the maximum significant carbohydrate accumulations after 65 DAE could be observed by applying the combined applications of (250 kg N ha^− 1^+ 400 g FKH 100 L^− 1^ water- T9 under 80% of ETc), although that were significantly equaled by applying combined applications of (250 kg N ha^− 1^+ 200 g FKH 100 L^− 1^ water- T8 under 100% of ETc). After 120 DAE, the maximum significant carbohydrate accumulations could be observed by adopting 80% of ETc and applying either the combined applications of (250 kg N ha^− 1^+ 200 g FKH 100 L^− 1^ water- T8) or (250 kg N ha^− 1^+ 400 g FKH 100 L^− 1^ water- T9).

The results in (Fig. 5D) indicated that by comparing irrigation levels in control treatment after 120 DAE, carbohydrate contents enhanced by (240.2 and 73.7%) for 100 and 80% of ETc irrigation levels compared to 60% of ETc, respectively. Meanwhile, relative to control 60% of ETc after 120 DAE in (Fig. 5F), leaves soluble sugars attained increases by 5.4 and 11.4% for 100 and 80% of ETc irrigation levels, respectively.

Concerning soluble sugars, the results in (Fig. 5E) showed that the maximum significant soluble sugars after 65 DAE could be obtained by applying the combined applications of (250 kg N ha^− 1^+ 400 g FKH 100 L^− 1^ water- T9 under 60% of ETc), although that were significantly equaled by applying combined applications of (250 kg N ha^− 1^+ 200 g FKH 100 L^− 1^ water- T8 under 60% of ETc). After 120 DAE (Fig. 5F), the maximum significant soluble sugar accumulations could be observed by adopting 100% ETc and applying the combined applications of (250 kg N ha^− 1^+ 400 g FKH 100 L^− 1^ water- T9).

### Carrot yield and WUE

**Fig. 6:**
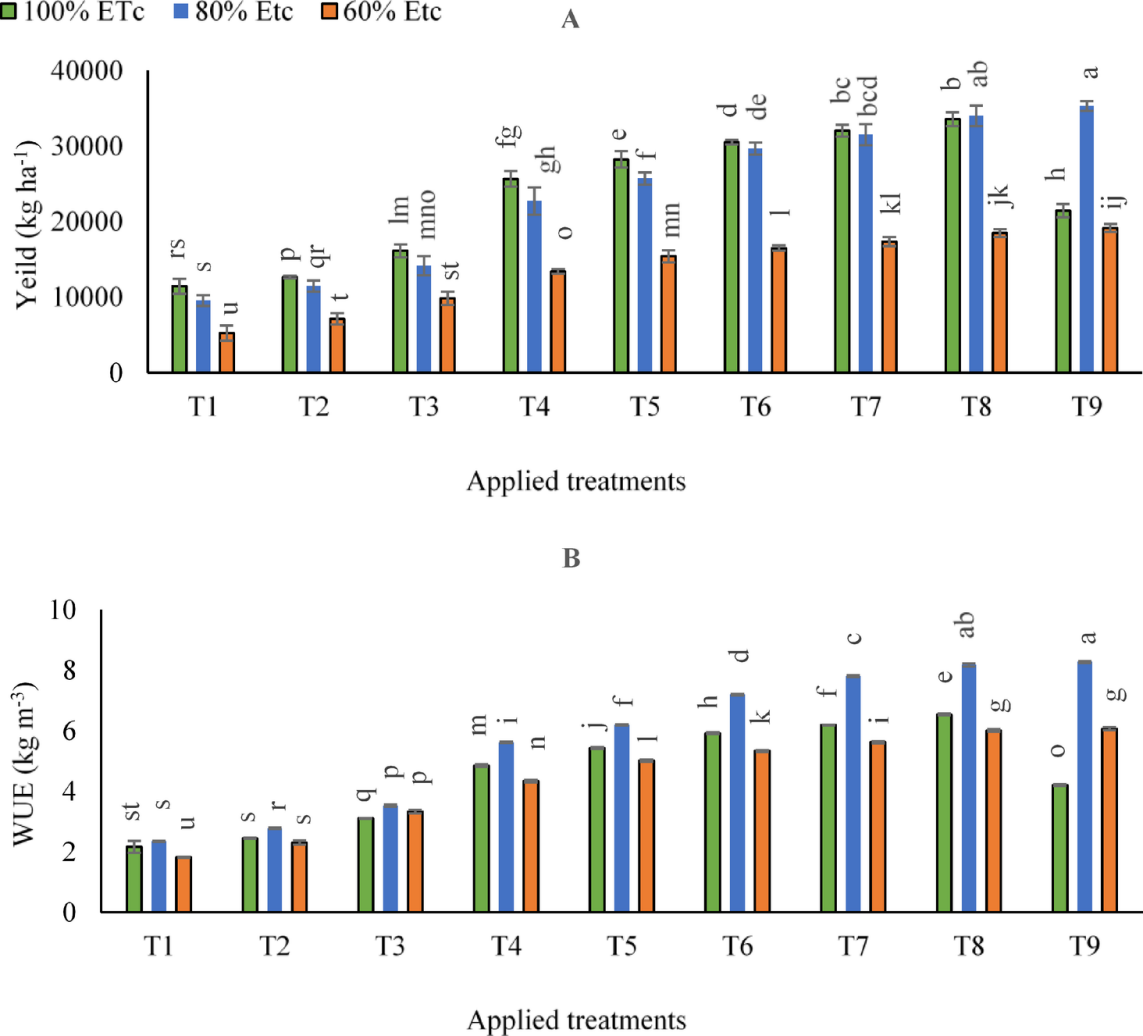
The mean interactive impacts of irrigation water levels and different applications and rates of [soil ammonium nitrate (SAN) and foliar potassium humate (FKH)] as individual or combined applications during the growing seasons (2019/2020 and 2020/2021) on (**A**) Carrot yield and (**B**) Water use efficiency (WUE). Error bars indicate standard errors from the mean. Bars with different letters are statistically significant at *p* ≤ 0.05. Abbreviations: T1 (tap water applications) (as control), T2 (SAN = 200 kg N ha^− 1^), T3 (SAN = 250 kg N ha^− 1^), T4 (FKH = 200 g 100 L^− 1^ water), T5 (FKH = 400 g 100 L^− 1^ water), T6 (SAN + FKH = 200 kg N ha^− 1^+ FKH = 200 g 100 L^− 1^ water), T7 (SAN + FKH = 200 kg N ha^− 1^+ FKH = 400 g 100 L^− 1^ water), T8 (SAN + FKH = 250 kg N ha^− 1^+ FKH = 200 g 100 L^− 1^ water), and T9 (SAN + FKH = 250 kg N ha^− 1^+ FKH = 400 g 100 L^− 1^ water).

According to the statistical analysis in (Supplementary Table S1) and (supplementary Table S6), the impacts of various irrigation levels and fertilized treatments (T_1_:T_9_) on carrot yield and WUE contents at 65 and 120 DAE were significant (*p* < 0.05). As can be observed in (Fig. 6A), by comparing the impacts of examined irrigation levels in the control treatment, the results demonstrated that the carrot yield was decreased by adopting 60% ETc irrigation level compared to 100% ETc by 54.2%, but, no marked significant differences were observed between 100 and 80% ETc. Hence, treating stressed carrot plants with SAN and FKH applications could help the plants overcome the negative impacts of water deficit and enhance carrot root yield under these circumstances. The results showed that by adopting 80% of ETc irrigation level, the highest carrot root yield was observed by applying combined applications of (250 kg N ha^− 1^+ 400 g FKH 100 L^− 1^ water- T9). However, that was significantly equaled by adopting the same irrigation level and applying a combined application of (250 kg N ha^− 1^+ 200 g FKH 100 L^− 1^ water- T8). On the other hand, it is worth noting that there was an antagonistic relationship between the obtained carrot root yield and higher application rates of SAN and FKH, and the intensity of this relationship was found to depend on the irrigation level implemented. In this sense, there was a gradual increase in irrigation amounts and application rates. Under (100% of ETc) irrigation level, the combined applications of (250 kg N ha^− 1^+ 400 g FKH 100 L^− 1^ water- T9) were significantly decreased compared to T6, T7, and T8 by 29.8, 33.1, and 36.1%, respectively.

Data depicted in (Fig. 6B) showed that the maximum rise of WUE was observed by adopting combined applications of (250 kg N ha^− 1^+ 200 g FKH 100 L^− 1^ water- T8) or by applying (250 kg N ha^− 1^+ 400 g FKH 100 L^− 1^ water- T9) under 80% of ETc irrigation level.

## Discussion

The primary agronomic practices in crop management revolve around the supply of water and nutrients through irrigation and fertilizer applications. These practices are particularly crucial under stressful conditions^64,65^. When addressing the management of proper irrigation and fertigation to enhance the growth of drought-sensitive crops such as carrots, it is imperative to conduct a comprehensive study on the impact of water deficit on crop growth in the short and long term, as well as to assess the potential yield if SAN and FKH are utilized. These applications have the potential to bolster plant resistance against the adverse effects of water deficit and represent a significant strategy for increasing WUE^66^.

### Carrot physiological and biochemical responses under full and limited irrigation in a short-term duration

Abiotic stresses such as water stress confine crop production and change their physiological and biochemical processes^67,68^. The current study investigated the impact of various water levels on carrots based on different physiological and biochemical characteristics. Based on the conclusions obtained in the short water deficit term (after 65 DAE), in the presence of inadequate water quantities (80 and 60% ETc), carrot plants exhibit low [pigment contents (except for carotenoids in plants irrigated by 80% of ETc), NH_4_, dry matter, and carbohydrate contents], alongside with high contents of [proline, H_2_O_2_, antioxidants, soluble sugar, and NO_3_ levels]. These influences on carrot traits by water deficit have also been reported by^69^. When carrot plants are subjected to water deficit, a lot of the physiological processes are directly or indirectly affected by chlorophyll contents. Previous studies have found that the pigment contents in the leaves decreased sharply with increasing water deficit^7,70^. Regarding the reduction in chlorophyll a and b content under water deficit, an explanation was represented by Mibei et al.^71^ attributed that due to stoma closure, leaf area reduction, gas exchange restriction, destruction of the pigment-protein complexes, and chloroplast lipids induced by oxidative; which ultimately declining photosynthetic pigments and activity. However, other studies have found mild water stress increases chlorophyll contents^72^. This could be associated with plants’ water stress tolerance, which can vary widely depending on plant ability, species, genotype, and developmental stage^73^. Nonetheless, carotenoids, among many metabolites, have been reported to have the ability to enhance within plant tissues under certain levels of water stress. In this concern, Zhang et al.^74^ showed that water deficit is a vital factor influencing the biosynthesis and metabolism of carotenoids. Hence, under unfavorable conditions, some plants tend to regulate the accumulation of carotenoids, which play significant roles in plant stress resistance. The authors further clarified carotenoids’ role as antioxidants, previously known to eliminate free radicals and maintain the redox balance, and better plant response to water deficit circumstances^75^. Overall, based on study findings, in contrast to 60% of ETc, this strategy (carotenoids enhancement) at 80% of ETc was adopted effectively for mitigating ROS accumulations (H_2_O_2_), possibly due to the effects of drought stress intensity. Moderate stress situations improve the amount of secondary metabolites in vegetables.

On the other hand, severe water stress causes negative changes in dry matter accumulation by affecting photosynthates and reductions in phosphate absorption since dry matter content is related to phosphate availability and uptake^76^. Plants experience reduced nutrient absorption and synthesis due to inadequate water supply^77^. On the other hand, it was reported that the synthesis, hydrolysis, and translocation of carbohydrates (polysaccharides) within leaf tissues are adversely affected by water deficit^78^. Under stress environments, plants adapt to these conditions, usually by physiological and biochemical modifications via stimulation of osmoprotectants and defensive antioxidants to confront the drought-generated hazard molecules^79^. In this experiment, carrot plants in the short water deficit term employed mechanisms to protect leaf tissues from the higher levels of ROS, including antioxidants and proline accumulations, sufficient accumulation of soluble sugar amounts in cytoplasm tissues, enhanced NO_3_ accumulation, and maintained carotenoid levels as much as possible, which matched with^69^. In this concern, Mibei et al.^71^ indicated that when plants are subjected to water deficiency, they produce huge amounts of antioxidants to adapt to the stress circumstances. A greater antioxidant production under these conditions helps carrot plants provide multiple benefits, including enhancing osmotic potential, balancing cell redox status, and maintaining cell membranes^80,81^. These changes ultimately resulted in increases in water uptake and cellular molecules preservation. Similarly, sustaining water status within plant tissues is vital to pass these conditions under unfavorable water deficit conditions. Hence, sufficient accumulations of soluble sugar and proline under stressful conditions decreased the water potential of the cells, promoted water retention in plants, and sustained adequate metabolic C/N ratios, in agreement with previous studies^73,82,83^. On the other hand, significant improvements were observed in leaf NO_3_ accumulations in plants subjected to limited irrigation and leaf NH_4_ accumulations under full irrigation levels. These results are in contrast with previous studies that demonstrated water deficit significantly reduced NO_3_ absorption and accumulation compared to full-watered irrigation conditions, whereas NH4 absorption and accumulation significantly enhanced under water-stressed irrigation conditions^84^. On the contrary, Tian et al.^85^ found increases in leaf NH_4_ contents and increases in leaf NO_3_ contents only when water deficit was accompanied by N deficiency. They added that NO_2_ contents in leaves were significantly increased under water deficit conditions. Also, Xia et al.^86^ mentioned that, in opposite trends to the roots, NO_3_ content in sweet potato leaves was significantly increased under water deficit (the same as in this study). In contrast, N-metabolism enzymes and nitrate reductase (NR) activities were significantly decreased. These conflicting findings between the previous studies could be due to the differences in the soil type, crop, and water stress intensity–duration–and frequency. On the other side, based on a study by Zia et al.^87^, plants under water stress conditions spend considerable energy dealing with stress tolerance, making this process a cost-intensive phenomenon. Urlić et al.^88^ showed that plants mostly use both N forms (NO_3_ and NH_4_); NO_3_ assimilation into plant metabolites requires more energy than NH_4_ because it has to be reduced. Therefore, under well-watered growth conditions, NH_4_ accumulations were expected to dominate, opposite to stress conditions. The reasons for the increase in NO_3_ accumulations under water deficit levels can be explained by (i) enhancement in the NO_3_ accumulations compensated osmotically for reductions in carbohydrate concentration when the photosynthesis rate was decreased. In this concern, similar to previous studies, a significant negative correlation was found between carbon (carbohydrate) and NO_3_ in plant tissues, which indicated that the plants used organic solutes instead of NO_3_ as part of iso-osmotic control^88,89^. Additionally, (ii) the reductions in NR activities under stressful conditions, which is considered the key enzyme of the transformation of NO_3_ into organic N compounds^90,91^. Finally, (iii) less availability and absorption of NH_4_ due to the alkalinity conditions and increased N losses via volatilization. In contrast to NO_3_ availability in the soil, NH_4_ availability and uptake are more tightly linked to soil pH and soil moisture alterations. In this respect, Jones et al.^92^ reported that when the soil pH exceeded 7.5, ammonia (NH_3_) began to volatilize, and the rate of this process doubled with increasing temperature. This increase in volatilization is due to the reduction in NH_3_ solubility in the soil solution as temperature rises^93^. Additionally, Stremińska and Błaszczyk^94^ investigated the impact of pH on NH_3_ loss through denitrification and found that the loss was most pronounced within a pH range of 6 to 8.

### Carrot physiological-biochemical responses under full and limited irrigation in the long-term duration

As Reid and Gillespie^95^ and Khalid et al.^96^ mention, carrots are very sensitive to water deficits. Nonetheless, they can tolerate moderate deficit levels^97,98^.

Under prolonged water stress, the equilibrium between ROS production and scavenging by antioxidants (including carotenoids) is disrupted, resulting in an imbalance in cellular redox homeostasis and more membrane damage^99,100^. In this concern, increased carotenoid production was effective at 80% of ETc irrigation level during the short-term period of water deficit, as evidenced by the illustrated figures and tables. Nevertheless, the findings of this study recorded a marked reduction in the impacts of water deficit that appeared during the long-term period on the carotene accumulations of carrots in both irrigation levels 80 and 60% of ETc. Water deficit sharply affected carotenoid production and accumulation at the end stage of plant growth^5,100^. An explanation was represented by Mibei et al.^71^ in this regard; they mentioned that although carotenes are a crucial part of the plant antioxidant defense system, they are very susceptible to oxidative destruction. Hence, in severe stress or prolonged water deficit, β-carotene could be rapidly destroyed. Consequently, they are no longer available to protect plants against oxidative damage.

The results obtained in this study showed that in the long term, carrot plants responded dynamically by enhancing their tolerant strategy by controlling soluble sugar synesis and carbohydrate hydrolysis and scavenging the ROS by proline and enzymatic components.

The increment of ROS species under stressed conditions accompanied other reactive nitrogen species (RNS) increments. Consequently, more damage is expected to disrupt the functioning of the plant cells, especially under 60% of ETc. In this regard, Deng et al.^101^ showed that higher RNS accumulations could exacerbate more damage for plants compared to ROS accumulations by triggering free radical peroxidation. In the current study, NO_3_^−^ contents were lower under fully-watered conditions (ETc 100%), as reported in lettuce^102^. They also added that ETc 80% was similar to ETc 100 for recording the same lower NO_3_ values, in contrast to ETc 60%. The results showed that in stressed carrot plants, a continuous enhancement in NO3 contents was noted before the harvest, representing a conservative plant strategy for water reduction. However, compared to stressful irrigation levels during the short term, NO_3_ contents in plant leaves tend to be lower. In agreement with this result, Sørensen et al.^103^ indicated that while high NO_3_ levels were found when water stress occurred during the early growth of carrots, the opposite was found when water stress occurred just before harvest. Similarly, Schlering et al.^76^ found a similar result on radish; they attributed these reductions in NO_3_ at harvest under water stress to the high radiation and evapotranspiration rates that might have resulted in balanced conditions between the uptake and assimilation of NO_3_. Although NO_3_ accumulations in leaves are considered lower RNS harmful, their presence was considered an indicator of increasing the accumulation of the other RNS species, especially NO, which is the end product of the NO_3_-NO_2_-NO pathway^104^. The presence of these components has conflicting impacts depending on their concentrations. It is beneficial under low levels or can cause oxidative damage to plant tissues when their production is enhanced more than cellular scavenging^105,106^. A study by Zhang et al.^107^ reported that the highest NO_3_ content in plant organs is unfavorable and even unacceptable in food nutrition. Obidiegwu et al.^108^ showed that leaf NO_3_ is vital in enhancing plant growth, improving osmotic regulation, and controlling stomatal closing by affecting guard cell depolarization. Perlikowski et al.^109^ reported that even lower RNS levels might decrease plants tolerant to water stress and their ability to recover after stress cessation. On the other hand, Umar and Iqbal^110^ have reported that NO_3_ level is negatively correlated with soluble carbon compounds, mainly sugars. The previous compounds (NO_3_ and soluble sugars) play complementary roles in cell turgor maintenance^111^. In the same concern, Seginer et al.^112^ hypothesized that plants regulate a mechanism to adjust the NO_3_ content to the soluble, non-structural carbon compound level for better osmotic turgor adjustments.

As stated in the short term, carbohydrate hydrolysis benefits carrot plants during irrigation-limited conditions due to the water-balancing mechanism. In keeping with this trend, the data from the study showed that at ETc 80% level, carbohydrate amounts such as (starch, glycogen, and cellulose) significantly decreased than ETc 100%. In contrast, soluble sugar amounts such as sucrose, glucose, and fructose increased. While at ETc 60% irrigation level, both carbohydrates and soluble sugars were decreased during the long-term compared to those observed at ETc 100%. In this experiment with irrigation at ETc 80%, the root activity rate, including absorption and exudates, probably increased more than the shoot activity rate, including the assimilation process and respiration, leading to a better plant tolerance against water deficiency. This situation attained better access to water and nutrients in soil layers for carrot roots. Also, the ability of the plant to transport the products of synthesis and hydrolysis processes from the source (leaves) to the sink (roots) is decreased slightly because of a higher increase in sap viscosity^113^. Similar results were reported by Lemoine et al.^114^ under mild water deficit levels, where plant shoot growth and photosynthetic productivity are restricted while both (root growth and development) continue and, consequently, the carbon flow to various sink organs (roots) is decreased. In this case, the accumulation of soluble sugar in leaves is increased, causing a significant reduction in water potential and enhancements in plants tolerant to water stress. Following this finding, studies by Lemoine et al.^114^ and Burke^115^ indicated that in high-respiration conditions (water stress conditions), soluble sugar accumulation in the plant leaves (source) increased, which has been assumed to (A) provide an energy source to maintain cell survival. (B) potential role in osmotic adjustment to sustain metabolic activity in plant leaves. Another hypothesis was represented by Hummel et al.^116^ that the sugar accumulation amounts in leaves may also be attributed to a reduction in carbon demands due to growth limitation. They suggested that water deficit conditions did not lead to carbon depletion, whereas they consistently observed a more favorable carbon balance.

When water stress intensity is increased more to 60% of ETc, the reductions of soluble sugar accumulation exceed those of unaffected plants at 100% of ETc. This study attributed these results to the impacts of adopting this irrigation level, which remarkably decreased root efficiency and increased nutrient fixation, leading to a decrease in P absorption, nutrient transport, respiration rate, and a decrease in new photoassimilates, resulting in notable reductions in the soluble sugar accumulation in leaves. This is supported by previous studies^117,118^.

Apart from the plant mechanisms discussion under short and long-water deficit terms, a significant reduction in carrot yield subjected to limited irrigation at 80& 60% of ETc was observed. However, in contrast to 60% ETc, these reductions in yield weren’t reflected in attaining significant decreases in WUE under 80% ETc irrigation level. This indicates that at 60% ETc, the available water for the plants is critically decreased, resulting in exceeded plant ability to control the excessive production of ROS species. Consequently, these impacts result in decreased root development and reduced WUE. This aligns with the findings of Ali^119^, which showed the sensitivity of carrot roots to water deficit.

Interestingly, when carrot plants were exposed to prolonged water stress at 80% ETc, positive impacts on carrot yield and WUE were observed. This highlights the importance of determining the suitable water stress pattern for carrot crops. It is concluded that at 80% ETc, although the carrot plants have to deal with prolonged water stress, the reduction in water application amounts stimulated root activities and efficiency. Moreover, this reduction in irrigation amounts promoted root penetration more profoundly into the soil layers, enabling increased root elongation. Consequently, water, nutrient uptake, assimilation process in the leaves, and accumulation of photoassimilates in the roots improved, resulting in enhanced yield and WUE under these circumstances.

Overall, the previous findings have clarified that the plant’s relative success in growing and developing well under water deficit conditions depends on water stress intensity and the plant’s ability to combine multiple adaptation strategies to survive where water stress happens. This could explain the beneficial impacts yielded 80% ETc compared to 60% ETc.

### Carrot responses to SAN and FKH applications under various irrigation levels in the short-term duration

Under stressful conditions, to improve plant tolerance to water deficit, it is essential to (i) adjust osmolyte accumulations, such as proline and sugar, to enhance osmoprotectants that help maintain cell turgor and water status and (ii) increase the level of antioxidants while controlling H_2_O_2_ production to improve tolerance efficiency. In this respect, SAN and FKH applications have the potential to attain that of plants affected by water stress. In the current study, significant enhancements were observed in the antioxidants, carbohydrates, and proline accompanied by substantial changes in NO_3_ and soluble sugar contents of carrot plants under short water stress-term conditions × high applications of SAN and FKH.

Comparing the changes in the various physiological and biochemical characteristics as a result of SAN and FKH applications with the control treatments indicated that after 65 DAE, the contents of (chlorophyll b, carotenoids except for 80% ETc, and dry matter) significantly increased by increasing the applied water and the examined application rates, and irrigation level bars took the same gradual increases patterns. Likewise, (H_2_O_2_ except for 60% ETc, proline, SOD, POD, and APX) significantly increased by decreasing the applied water and increasing application rates of SAN and FKH, and irrigation level bars took the same gradual patterns. Conversely, modifications in the obtained patterns were found in the contents of (chlorophyll a, NO_3_, NH_4_, CAT, carbohydrates, and soluble sugars), especially at 80% of the ETc irrigation level in the combination treatments. This may account for the impact of SAN and FKH applications in attaining better stress tolerance by these modifications under this irrigation level. These modifications are supposed to play a vital role in osmotic regulation in water stress conditions. They can affect various physio-biochemical processes related to plant defense functions, plant metabolism, and synthesis, either directly or indirectly.

The modifications in carbohydrate and soluble sugar contents highlight the potential role in osmotic adjustment under water deficit conditions. Increased accumulation of chlorophyll a in leaves under ETc 80% resulted in enhanced light harvesting, decreased chlorophyll fluorescence, and helped to convert the absorbed photons energy into chemical energy^120^. Similarly, increased CAT production in carrot leaves at 80% of the ETc protects plant cells from rising oxidative levels under these conditions, promoting the antioxidant capacity and improving plants’ ability to resist water deficiency; these findings agree with^121^. Among antioxidant enzymes, CAT is the most efficient and has the potential to neutralize ROS species, including H_2_O_2_, into H_2_O and O_2_^122^. In this respect, studies by Alam and Ghosh^123^ and Poli et al.^124^ have highlighted the efficient role of higher CAT activities and contents in scavenging the ROS at the early plant stages compared to the other antioxidant enzymes. Also, previous studies observed that sensitive crops like carrots were associated with decreased activities and contents of CAT under water deficit conditions^125,126^. These noteworthy results underscore the potential of SAN and FKH applications as valuable tools for bolstering carrot resistance to water deficiency by modifying and increasing CAT contents.

By adopting an ETc 80% irrigation level, the combinations of SAN and FKH applications in a short water deficit period effectively strengthen carrot plants’ ability to withstand irrigation water deficiency and protect them against the harmful effects of high levels of NH_4_ in leaves. In this respect, de Castro et al.^127^ concluded that a high concentration of NH_4_ in the plant leaves increased chloroplast degradation, decreased photosynthesis rate, and intensified NH_4_ toxicity. They attributed NH_4_ toxicity to (i) the lack of balance between N absorption and assimilation and (ii) the acidic stress induced by the higher proton amounts in the chloroplasts released during NH_4_ assimilation. Hence, reductions in NH_4_ concentrations have been previously observed by SAN and FKH application combinations under ETc 80% irrigation level, as opposed to (ETc 100 and 60%) and the individual SAN applications. This study, therefore, concluded that NH4 regulation in plants is linked to (i) the obtained irrigation level and (ii) appropriate application combined with SAN, which is essential in higher plants to balance NH_4_ and NO_3_ in N assimilation^107,128^. Conversely, controlling NO_3_ accumulation by SAN and FKH combinations at ETc 80% irrigation level and equalizing or reducing their concentration in leaves compared to ETc 100% helps adapt carrot plants to abiotic stress.

These modifications show carrot plants’ dynamic responses to short-water deficits and highlight the fundamental roles of chlorophyll a, NO_3_, NH_4_, carbohydrates, soluble sugars, and CAT in regulating and improving different physiological processes that allow plants to adapt and survive in diverse conditions.

### Carrot responses to SAN and FKH applications under various irrigation levels in the long-term duration

As indicated in the short-water deficit term, the simultaneous application of SAN and FKH improved soluble sugar contents compared to the control treatment, especially in the limited irrigation levels, compensating for the declined water stress tolerance efficiency. This resulted in higher levels of osmoprotectants in carrot leaves under these conditions, leading to enhanced physiological processes and water potential, ultimately alleviating water stress’s harmful impacts. The results showed that these adaptive responses continued, and the converted amounts of soluble sugar exceeded or equaled those obtained under complete irrigation treatment by applying SAN and FKH under ETc 80 and 60% of ETc, except for T9. The reductions in soluble sugars under ETc 80 and 60% of ETc as a consequence of SAN and FKH applications are due to the effective antioxidant system activation, led to a decreased need for soluble sugar accumulations at this point, which is ultimately contributed to raising the carbohydrate synthesis processes.

Another differential plant response examined in this study was the impact of SAN and FKH applications on pigment contents under various irrigation levels. To mitigate the detrimental effects of water deficit on chlorophyll contents, individual and combined applications of SAN and FKH were applied. The findings of the current study indicated that these applications under water deficit levels had a significant positive impact on chlorophyll contents in carrots, which is consistent with the findings of other studies on the positive effects of SAN and FKH applications under mild water deficiency on similar plants^43,129,130^. However, under ETc 80%, the impact of these applications was found to be more significant on chlorophyll a and chlorophyll b content, which equaled or passed ETc 100%. In this concern, Khayatnezhad & Gholamin^131^ and Chowdhury et al.^132^ reported plants that can sustain higher chlorophyll contents under water deficit conditions can contribute to avoiding negative influences and be more resistant to stress. Under study conditions, this has been achieved by using these applications. These increases in chlorophyll concentration under ETc 80% are attributed to (SAN and FKH) ability to promote chlorophyll formation while concomitantly preventing its degradation, which aligns with previous research conducted by^133^. Under biotic or abiotic stresses, the application of both N and humic substances showed significant improvements in plant pigments^134,135^. Additionally, humic materials containing potassium have dual positive effects on plant growth, as both humate and potassium are stress inducers^136^. Moreover, this positive impact on chlorophyll concentrations can be attributed to the irrigation level it adopts, which improves root uptake efficiency and facilitates plant assimilation processes. Understanding this plant response to SAN and FKH applications under mild water deficiency can help scientists and farmers develop innovative practices for alleviating the detrimental impacts and enhancing the tolerance of carrot plants under challenging water deficit conditions. On the other hand, a notable increase in carotenoid content was observed when SAN and FKH applications were applied in water-stressed carrot plants compared to the control treatment. These elevated levels of carotenoids indicate the presence of oxidative damage in carrot plants experiencing water stress. The elevated levels of ROS/RNS (NO_3_ and H_2_O_2_) under water stress resulted in harmful conditions termed cell redox status, which exceeded the ability of carotenoids to deal with loneliness.

Regarding NO_3_ and H_2_O_2_ levels under water scarcity, the results indicate that the different water levels and the SAN and FKH applications highly influenced NO_3_ concentrations in carrot leaves. Based on the measured data during the long-term period, under 80&60% of ETc, high NO_3_ concentration in carrot leaves was observed with the individual applications and rates of SAN and KH compared to ETc100%. In contrast, the combination of SAN and FKH significantly decreased NO_3_ concentrations under ETc 80%, compared to ETc100%. While under ETc 60%, there was a marked increment in NO_3_ by applying the combined applications. However, these increments didn’t exceed or equal those obtained in ETc100% irrigation levels. Zhang et al.^107^ suggested that NO_3_ increases under full irrigation conditions could happen mainly due to the imbalance between N supply and demand, which is required for optimal growth. Moreover, Anjana & Iqbal^137^ found that externally higher applied K rates help to enhance NO_3_ absorption and transport toward the plant leaves under normal conditions. Additionally, numerous studies have highlighted the beneficial impacts of foliar applications of SAN and humic components on various crops El-Nasr^138^ and Tavares et al.^139^. They found a strong association between these examined applications and NO_3_, resulting in the enhancements of NO_3_ accumulation. However, in this regard, Nazaryuk et al.^140^, Fawzy^141^, and Shahein et al.^142^ found that applying humic acid as a foliar spray gave lower NO_3_ concentrations in head lettuce. At the same time, Haghighi et al.^143^ indicated no significant impact of humic acid on NO_3_ accumulation in tomatoes.

On the other hand, we need to advance our understanding of the mechanisms by which NO_3_ production is regulated under water deficit conditions based on stress intensity and duration. It is necessary to remember the significant role that NO_3_ plays in enhancing plant development and osmotic regulation^144^. Consequently, under severe water stress (ETc 60%), higher NO_3_ amounts could effectively improve plant tolerance, which, in turn, is promoted by the combined application of SAN and FKH applications. Meng et al.^145^ concluded another possible reason for improved NO_3_ content in leaves under water deficit levels was closely related to increased K uptake and transport under these conditions. Besides that, under water deficit conditions, the oxidative process was amplified by reducing CO_2_ assimilation, which induces excess excitation energy and electron flux to O_2_^146^.

These reductions can be achieved by applying SAN and FKH under ETc 80%. This study postulated that under the ETc 80% level, carrot plants experience a certain degree of water stress, which triggers some physiological and biochemical responses in higher NO_3_ amounts. Stressed carrot plants can benefit from applying SAN and FKH, as these can help activate their defense mechanisms. This can improve the plant’s water status and synthesis processes by increasing nutrient uptake while reducing the harmful impacts of ROS (H_2_O_2_ levels). Consequently, this result concluded that the assimilation of NH_4_ was promoted under these conditions accompanied by decreasing NO_3_ amounts. Therefore, Bian et al.^91^ highlighted the potential role of the optimal irrigation regime in decreasing NO3 content and concomitantly attaining water-saving, which could provide an effective strategy under water deficit conditions.

On the other hand, under such growth conditions, the adoption of SAN and FKH applications at ETc 100 & ETc 80 irrigation levels increased antioxidant accumulation of (CAT, POD and, APX) and proline, which is due to improved nutrient uptake levels like N and iron. At the same time, Wang et al.^147^ demonstrated that iron is considered the main component in the formation of CAT, POD, and APX. Previous studies have indicated that N is involved in proline biosynthesis and differentiation across the plant growth cycle^148–150^. In the same context, Meena et al.^150^ stated that phytohormones regulate proline metabolism by controlling the accumulation of N_2_ or calcium (Ca^2+^). Nonetheless, the current study observed significant changes in carrot leaves’ antioxidants and proline contents under (SAN and FKH) applications × long water stress-term conditions. In contrast to the short water stress term, which attained high content, the collective antioxidants and proline values were lower in the long water stress period. However, ETc 100 & ETc 80 irrigation levels were taking the same gradual pattern as the short water stress term (ETc 80 > ETc 100); the ETc, 60% level, was the exception. These collective reductions in antioxidants and proline levels highlight the dynamic nature of plant responses to water deficit and the positive impacts of applications like SAN and FKH in improving growth conditions, as mentioned by a previous study^151^. Meena et al.^150^ illustrated that the catabolic activity and proline contents start to decrease within plant tissues when there is no need or their contents are in excess amounts. Nevertheless, this study attributed these declines in the long-term period to the reductions in water and nutrient uptake capacity^152^, which decline with root aging. Simultaneously, the biosynthesis of antioxidants and proline decreases under these conditions, especially under the ETc 60% level.

### Impacts of SAN and FKH applications under various irrigation levels on carrot yield and WUE

Conclusively, the findings in the current study showed that the adoption of combined applications like T8 or T9 under ETc by 80% increased the carrots yield and achieved a higher WUE. In this respect, this study concluded that by applying these combined applications and rates under this limited irrigation level, many effective mechanisms discussed earlier, including regulation of NH_4_ and NO_3_, enhanced pigment contents, improved antioxidant defense systems, and sufficient proline concentrations in the cytoplasm, seem to have yielded beneficial effects throughout the short and prolonged water stress until harvest, which is consistent with findings from^43^. These mechanisms, accompanied by other improvements in nutrient homeostasis, plant growth, and development, enhance their drought tolerance and overall yield under such growth conditions.

Under stressful conditions at ETc 60%, plants produce ROS species, which help maintain carrot growth and enhance stress tolerance. Nonetheless, by increasing stress intensity, the ROS production rate increases exponentially, exceeding the ability of antioxidant scavengers. Hence, the plants, especially those sensitive to water deficit, cannot control and detoxify reactive oxygen species within plant cells, leading to detrimental oxidative consequences in plant development, yield, and WUE. Therefore, the results indicated that irrespective of adopting individual and combined applications of SAN + FKH, the lowest carrot yield and WUE were achieved under ETc 60%.

On the other hand, one interesting finding in this study was the sudden reduction in yield and WUE by applying the higher rate of SAN and FKH in treatment T9 under the ETc 100% level. Such a finding is significant as adopting this rate under ETc 100% is supposed to attain the highest improvements in carrot yield relative to the low applications. In this respect, this study hypothesized two reasons: A) that can be attributed to the change in C/N and N/P ratios. This could be confirmed through the nutritional analysis. This led to an increase in the vegetative growth rate processes (aerial parts) rather than the reproductive processes, which is reflected in decreased or delayed accumulation processes and led to unfavorable outcomes on yield. In this regard, a study by^153^ found a similar result in sweet potatoes by applying a higher application rate under well-watered irrigation conditions. The other reason, B), can be attributed to the observed increments in NH_4_ concentrations in the leaves. That triggered a series of negative physiological impacts on chlorophyll contents. As demonstrated previously, a high concentration of NH_4_ has deleterious consequences on chloroplasts and photosynthesis rate^127^. Also, in support of these findings, Xia et al.^86^ addressed the possible reasons behind the superiority of mild irrigation level compared to whole irrigation level in terms of groundnut yield to: (i) improve nitrification intensity under mild irrigation level, resulting in increased N available contents, maintained photosynthetic components, and enhanced nutrient transport and accumulate to sink organs; (ii) the presence of some reductions in chlorophyll content and plant tolerance under excessive applications of irrigation and N.

## Conclusion

The findings indicate that in the short water stress term duration, by irrigating plants at 80% of crop evapotranspiration, carrot plants tend to increase the synthesis of (carotenoids except for 60% of crop evapotranspiration, nitrate, proline, antioxidants, and soluble sugar) and decrease (ammonium and carbohydrate contents) in response to the production of reactive oxygen species. In the prolonged water stress term duration, carrot plants tend to modify their defense mechanisms by increasing (nitrate, proline except for 60% of crop evapotranspiration, antioxidants except for 60% of crop evapotranspiration, and soluble sugar) and decreasing ammonium and carbohydrate contents. These adaptive responses at 80% of crop evapotranspiration improve their resilience to water deficiency and allow carrot plants to maintain physiological processes and yield. By rearranging chlorophyll a, nitrate, ammonium, catalase, carbohydrate, and soluble sugars accumulations at 80% of crop evapotranspiration, the combined application of ammonium nitrate and potassium humate helps plants to optimize their adaptive responses and minimize the detrimental impacts of water deficit. Overall, the findings indicate that ammonium nitrate and potassium humate applications and rates positively affected carrot physiological and biochemical processes and yield under various irrigation levels despite observed negative impacts at the high rates of these applications under full irrigation level. Therefore, it is recommended to apply combined applications of ammonium nitrate at 250 kg N ha^− 1^ in combination with foliar potassium humate at 200 g 100 L^− 1^ water under 80% of the crop evapotranspiration for carrots to improve chlorophyll content, antioxidant enzymes, proline, carbohydrate, yield and water use efficiency of the carrot plants. This approach highlights the potential benefits of combining nutrient applications and irrigation management strategies to enhance carrot performance and address the challenges of water scarcity in agriculture.

## Electronic supplementary material

Below is the link to the electronic supplementary material.

Supplementary Material 1

## Data Availability

All the data related to this work can be sourced from the corresponding authors.
