# Supplementary material for: Synergistic effect of ammonium and potassium on carrot growth, physio-biochemical mechanisms, and water use efficiency under varying irrigation regimes
Source: Sci Rep. 2025 May 9;15:16151. doi: 10.1038/s41598-025-00690-3 (PMC12062451; doi:10.1038/s41598-025-00690-3)
Supplement: Supplementary file 1 — Supplementary Material 1 [file 41598_2025_690_MOESM1_ESM.docx]

**Supplementary Table S1** Variance analysis of the investigated parameters

| **Source of variation** | **df** | **CHA**  **65** | **CHA**  **120** | | **CHB**  **65** | **CHB**  **120** | **CARO**  **65** | **CARO**  **120** | **CAT**  **65** | **CAT**  **120** | **POD**  **65** | **POD**  **120** |
| --- | --- | --- | --- | --- | --- | --- | --- | --- | --- | --- | --- | --- |
| Irrigation water levels (IR) | 2 | ***** | ***** | | * | * | * | * | * | * | * | * |
| Fertilization treatments (FT) | 8 | * | * | | * | * | * | * | * | * | * | * |
| IR × FT | 16 | * | * | | * | ***** | * | NS | * | * | * | * |
|  | ***p*-value** | | | | | | | | | | | |
|  |  | <0.001 | <0.001 | | <0.001 | <0.001 | <0.001 | <0.001 | <0.001 | <0.001 | <0.001 | <0.001 |
|  |  | <0.001 | <0.001 | | <0.001 | <0.001 | <0.001 | <0.001 | <0.001 | <0.001 | <0.001 | <0.001 |
|  |  | <0.001 | <0.001 | | <0.001 | <0.001 | <0.001 | 0.09 | <0.001 | <0.001 | <0.001 | <0.001 |
|  |  | **NH_4_**  **65** | **NH_4_**  **120** | | **SOD**  **65** | **SOD**  **120** | **APX**  **65** | **APX**  **120** | **NO_3_**  **65** | **NO_3_**  **120** | **H_2_O_2_**  **65** | **H_2_O_2_**  **120** |
| Irrigation water levels (IR) | 2 | * | * | | * | * | * | * | * | * | * | * |
| Fertilization treatments (FT) | 8 | * | * | | * | * | * | * | * | * | * | * |
| IR × FT | 16 | * | * | | * | * | * | * | * | * | * | * |
|  | ***p*-value** | | | | | | | | | | | |
|  |  | <0.001 | <0.001 | | <0.001 | <0.001 | <0.001 | <0.001 | <0.001 | <0.001 | <0.001 | <0.001 |
|  |  | <0.001 | <0.001 | | <0.001 | <0.001 | <0.001 | <0.001 | <0.001 | <0.001 | <0.001 | <0.001 |
|  |  | <0.001 | <0.001 | | <0.001 | 0.002 | <0.001 | <0.001 | <0.001 | <0.001 | <0.001 | <0.001 |
|  |  | **PRO**  **65** | **PRO**  **120** | | **RY** | **CARB**  **65** | **CARB**  **120** | **SOLS**  **65** | **SOLS**  **120** | **DM**  **65** | **DM**  **120** | **WUE** |
| Irrigation water levels (IR) | 2 | * | * | | * | * | * | * | * | * | * | * |
| Fertilization treatments (FT) | 8 | * | * | | * | * | * | * | * | * | * | * |
| IR × FT | 16 | * | * | | * | * | * | * | * | * | * | * |
|  | ***p*-value** | | | | | | | | | | | |
|  |  | <0.001 | <0.001 | 0.002 | | <0.001 | <0.001 | <0.001 | <0.001 | <0.001 | <0.001 | <0.001 |
|  |  | <0.001 | <0.001 | <0.001 | | <0.001 | <0.001 | <0.001 | <0.001 | <0.001 | <0.001 | <0.001 |
|  |  | <0.001 | <0.001 | <0.001 | | <0.001 | <0.001 | <0.001 | <0.001 | <0.001 | <0.001 | <0.001 |

CHA 65: Chlorophyll a- at 65 DAE; CHA 120: Chlorophyll a- at 120 DAE; CHB 65: Chlorophyll b- at 65 DAE; CHB 120: CAR 65: Carotenoids at 65 DAE; CAR 120: Carotenoids at 120 DAE; CARB 65: Carbohydrate at 65 DAE; CARB 120: Carbohydrate at 120 DAE; SOLS 65: Soluble sugars at 65 DAE; SOLS 65: Soluble sugars at 65 DAE; H_2_O_2_ 65: Hydrogen peroxide at 65 DAE; H_2_O_2_ 65: Hydrogen peroxide at 65 DAE; RY: roots yield; CAT 65: catalase at 65 DAE; CAT 120: catalase at 120 DAE; SOD 65: superoxide dismutase at 65 DAE; SOD 120: superoxide dismutase at 120 DAE; POD 65: peroxidase at 65 DAE; POD 120: peroxidase at 120 DAE; APX 65: Ascorbate peroxidase at 65 DAE; APX 120: Ascorbate peroxidase at 120 DAE; NO_3_ 65: Nitrate in leaves at 65 DAE; NO_3_ 120: Nitrate in leaves at 120 DAE; NH_4_ 65: Ammonium in leaves at 65 DAE; NH_4_ 120: Ammonium in leaves at 120 DAE; PRO 65: Proline at 65 DAE; PRO 120: Proline at 120 DAE; DM: dry matter; WUE: water use efficiency; NS: non-significance; * significance at P ≤ 0.05.

**Supplementary Table S2** The individual effects of adopting irrigation levels and fertilization (ammonium nitrate and potassium humate) treatments on (chlorophyll a_,_ chlorophyll b, and carotenoids) in carrot leaves during the growing seasons of 2019/2020 and 2020/2021

| **Studied factors** | **Chlorophyll a (mg g^-1^ fw)**  **After 65 days of**  **emergence** | **Chlorophyll a**  **(mg g^-1^ fw)**  **After 120 days of**  **emergence** | **Chlorophyll b (mg g^-1^ fw)**  **After 65 days of**  **emergence** | **Chlorophyll b (mg g^-1^ fw)**  **After 120 days of**  **emergence** | **Carotenoids**  **(mg g^-1^ fw)**  **After 65 days of**  **emergence** | **Carotenoids**  **(mg g^-1^ fw)**  **After 120 days of**  **emergence** |
| --- | --- | --- | --- | --- | --- | --- |
| **Irrigation levels** |  |  |  |  |  |  |
| 100 (%) | 1.68a | 2.65b | 0.802a | 1.19a | 0.276b | 0.429a |
| 80 (%) | 1.58b | 2.72a | 0.659b | 1.16b | 0.447a | 0.348b |
| 60 (%) | 1.37c | 1.91c | 0.479c | 0.69c | 0.115c | 0.237c |
| **Fertilization treatments** |  |  |  |  |  |  |
| T1 | 1.21i | 2.1f | 0.535i | 0.868h | 0.197i | 0.132i |
| T2 | 1.33h | 2.26d | 0.553h | 0.913g | 0.220h | 0.173h |
| T3 | 1.44e | 2.36c | 0.612g | 0.973f | 0.238g | 0.210g |
| T4 | 1.37f | 2.16e | 0.652f | 1.02e | 0.253f | 0.263f |
| T5 | 1.36g | 2.27d | 0.661e | 1.06d | 0.274e | 0.337e |
| T6 | 1.66d | 2.61b | 0.683c | 1.08c | 0.298d | 0.390d |
| T7 | 1.74c | 2.65b | 0.713b | 1.10b | 0.320c | 0.463c |
| T8 | 1.84b | 2.70a | 0.740a | 1.11a | 0.349b | 0.507b |
| T9 | 1.94a | 2.72a | 0.673d | 1.01e | 0.365a | 0.567a |

Abbreviations: T1 (tap water applications) (as control), T2 (soil applications of ammonium nitrate = 200 kg N ha^-1^), T3 (soil applications of ammonium nitrate = 250 kg N ha^-1^), T4 (foliar applications of potassium humate = 200 g 100 L^-1^ water), T5 (foliar applications of potassium humate = 400 g 100 L^-1^ water), T6 (soil applications of ammonium nitrate + foliar applications of potassium humate = 200 kg N ha^-1^+ foliar applications of potassium humate =200 g 100 L^-1^ water), T7 (soil applications of ammonium nitrate + foliar applications of potassium humate = 200 kg N ha^-1^+ foliar applications of potassium humate = 400 g 100 L^-1^ water), T8 (soil applications of ammonium nitrate + foliar applications of potassium humate = 250 kg N ha^-1^+ foliar applications of potassium humate = 200 g 100 L^-1^ water), and T9 (soil applications of ammonium nitrate + foliar applications of potassium humate = 250 kg N ha^-1^+ foliar applications of potassium humate = 400 g 100 L^-1^ water)

**Supplementary Table S3** The individual effects of adopting irrigation levels and fertilization (ammonium nitrate and potassium humate) treatments on (H_2_O_2,_ NO_3_, NH_4_, and proline) in carrot leaves during the growing seasons of 2019/2020 and 2020/2021

| **Studied factors** | **Leaf H_2_O_2_**  **(μmol g^−1^ fw)**  **After 65 days of**  **emergence** | **Leaf H_2_O_2_**  **(μmol g^−1^ fw)**  **After 120 days of**  **emergence** | **NO_3_**  **(mg g^−1^)**  **After 65 days of**  **emergence** | **NO_3_**  **(mg g^−1^)**  **After 120 days of**  **emergence** | **NH_4_**  **(mg g^−1^)**  **After 65 days of**  **emergence** | **NH_4_**  **(mg g^−1^)**  **After 120 days of**  **emergence** | **Proline**  **(mg g^−1^ fw)**  **After 65 days of**  **emergence** | **Proline**  **(mg g^−1^ fw)**  **After 120 days of**  **emergence** |
| --- | --- | --- | --- | --- | --- | --- | --- | --- |
| **Irrigation levels** |  |  |  |  |  |  |  |  |
| 100 (%) | 7.86c | 10.39c | 76.88a | 67.74b | 4.57a | 7.76a | 10.52c | 19.58b |
| 80 (%) | 12.67b | 15.73b | 73.05b | 67.82b | 3.78b | 5.40b | 14.03b | 20.27a |
| 60 (%) | 15.18a | 21.34a | 68.94c | 72.48a | 3.06c | 3.34c | 23.09a | 17.66c |
| **Fertilization treatments** |  |  |  |  |  |  |  |  |
| T1 | 11.50i | 15.69f | 61.93h | 61.09ef | 3.70e | 5.02h | 14.47i | 17.8i |
| T2 | 11.59h | 15.76e | 62.05h | 60.68f | 4.32b | 5.64d | 14.85h | 18.14h |
| T3 | 11.69g | 15.85d | 64.72g | 61.55e | 4.52a | 5.93b | 15.16g | 18.44g |
| T4 | 11.81f | 15.32g | 65.89f | 59.85g | 3.67ef | 4.98i | 15.56f | 18.85f |
| T5 | 11.88e | 15.71f | 67.22e | 60.94ef | 3.86d | 5.15g | 15.86e | 19.15e |
| T6 | 11.99d | 15.82d | 74.30d | 72.88d | 3.21h | 5.36f | 16.14d | 19.43d |
| T7 | 12.14c | 16.14a | 81.28c | 78.64c | 3.38g | 5.52e | 16.54c | 19.83c |
| T8 | 12.18b | 16.01c | 86.64b | 81.36b | 3.61f | 5.81c | 16.86b | 20.15b |
| T9 | 12.28a | 16.05b | 92.56a | 87.13a | 3.98c | 6.11a | 17.47a | 20.76a |

Abbreviations: H_2_O_2_: Hydrogen peroxide; NO_3_: Nitrate; NH_4_: Ammonium; T1 (tap water applications) (as control), T2 (soil applications of ammonium nitrate = 200 kg N ha^-1^), T3 (soil applications of ammonium nitrate = 250 kg N ha^-1^), T4 (foliar applications of potassium humate = 200 g 100 L^-1^ water), T5 (foliar applications of potassium humate = 400 g 100 L^-1^ water), T6 (soil applications of ammonium nitrate + foliar applications of potassium humate = 200 kg N ha^-1^+ foliar applications of potassium humate =200 g 100 L^-1^ water), T7 (soil applications of ammonium nitrate + foliar applications of potassium humate = 200 kg N ha^-1^+ foliar applications of potassium humate = 400 g 100 L^-1^ water), T8 (soil applications of ammonium nitrate + foliar applications of potassium humate = 250 kg N ha^-1^+ foliar applications of potassium humate = 200 g 100 L^-1^ water), and T9 (soil applications of ammonium nitrate + foliar applications of potassium humate = 250 kg N ha^-1^+ foliar applications of potassium humate = 400 g 100 L^-1^ water)

**Supplementary Table S4** The individual effects of adopting irrigation levels and fertilization (ammonium nitrate and potassium humate) treatments on the antioxidant enzymes in carrot leaves during the growing seasons of 2019/2020 and 2020/2021

| **Studied factors** | **CAT**  **(μmol min^-1^ mg^-1^)**  **After 65 days of**  **emergence** | **CAT**  **(μmol min^-1^ mg^-1^)**  **After 120 days of**  **emergence** | **POD**  **(μmol min^-1^ mg^-1^)**  **After 65 days of**  **emergence** | **POD**  **(μmol min^-1^ mg^-1^)**  **After 120 days of**  **emergence** | **SOD**  **(U mg^-1^)**  **After 65 days of**  **emergence** | **SOD**  **(U mg^-1^)**  **After 120 days of**  **emergence** | **APX**  **(μmol min^-1^ mg^-1^)**  **After 65 days of**  **emergence** | **APX**  **(μmol min^-1^ mg^-1^)**  **After 120 days of**  **emergence** |
| --- | --- | --- | --- | --- | --- | --- | --- | --- |
| **Irrigation levels** |  |  |  |  |  |  |  |  |
| 100 (%) | 27.18c | 29.23b | 24.77c | 22.47b | 0.689c | 0.355b | 2.38c | 2.59b |
| 80 (%) | 33.73a | 33.91a | 27.52b | 24.73a | 0.758b | 0.555a | 2.76b | 2.88a |
| 60 (%) | 32.17b | 22.23c | 34.08a | 17.60c | 0.937a | 0.271c | 3.22a | 2.12c |
| **Fertilization treatments** |  |  |  |  |  |  |  |  |
| T1 | 25.59i | 21.39i | 25.89i | 18.69i | 0.675i | 0.242i | 2.35h | 2.11g |
| T2 | 27.0h | 24.47h | 26.66h | 19.47h | 0.705h | 0.325h | 2.50g | 2.26f |
| T3 | 28.07g | 25.86g | 27.20g | 20.0g | 0.727g | 0.346g | 2.60e | 2.37de |
| T4 | 29.32f | 26.48f | 27.72f | 20.53f | 0.761f | 0.381f | 2.55f | 2.31ef |
| T5 | 30.97e | 28.11e | 28.36e | 21.20e | 0.790e | 0.409d | 2.64d | 2.41d |
| T6 | 32.41d | 30.55d | 29.61d | 22.41d | 0.818d | 0.400e | 2.87c | 2.61c |
| T7 | 33.74c | 32.24c | 30.19c | 23.02c | 0.858c | 0.432c | 3.03b | 2.74b |
| T8 | 36.29a | 34.05a | 31.31b | 24.11b | 0.896b | 0.515a | 2.99a | 2.99a |
| T9 | 35.86b | 32.97b | 32.19a | 24.99a | 0.924a | 0.492b | 3.01a | 3.01a |

Abbreviations: CAT: catalase; SOD: superoxide dismutase; POD: peroxidase; APX: Ascorbate peroxidase. T1 (tap water applications) (as control), T2 (soil applications of ammonium nitrate = 200 kg N ha^-1^), T3 (soil applications of ammonium nitrate = 250 kg N ha^-1^), T4 (foliar applications of potassium humate = 200 g 100 L^-1^ water), T5 (foliar applications of potassium humate = 400 g 100 L^-1^ water), T6 (soil applications of ammonium nitrate + foliar applications of potassium humate = 200 kg N ha^-1^+ foliar applications of potassium humate =200 g 100 L^-1^ water), T7 (soil applications of ammonium nitrate + foliar applications of potassium humate = 200 kg N ha^-1^+ foliar applications of potassium humate = 400 g 100 L^-1^ water), T8 (soil applications of ammonium nitrate + foliar applications of potassium humate = 250 kg N ha^-1^+ foliar applications of potassium humate = 200 g 100 L^-1^ water), and T9 (soil applications of ammonium nitrate + foliar applications of potassium humate = 250 kg N ha^-1^+ foliar applications of potassium humate = 400 g 100 L^-1^ water).

**Supplementary Table S5** The individual effects of adopting irrigation levels and fertilization (ammonium nitrate and potassium humate) treatments on (soluble sugar_,_ carbohydrate, and dry matter) in carrot leaves during the growing seasons of 2019/2020 and 2020/2021

| **Studied factors** | **Soluble sugar**  **(mg g^−1^ dw)**  **After 65 days of**  **emergence** | **Soluble sugar**  **(mg g^−1^ dw)**  **After 120 days of**  **emergence** | **Carbohydrate**  **(mg g^−1^)**  **After 65 days of**  **emergence** | **Carbohydrate**  **(mg g^−1^)**  **After 120 days of**  **emergence** | **Dry matter**  **(%)**  **After 65 days of**  **emergence** | **Dry matter**  **(%)**  **After 120 days of**  **emergence** |
| --- | --- | --- | --- | --- | --- | --- |
| **Irrigation levels** |  |  |  |  |  |  |
| 100 (%) | 76.36c | 92.00b | 153.5a | 186.3a | 16.45a | 22.72a |
| 80 (%) | 79.10b | 96.89a | 136.7b | 143.2b | 13.11b | 21.59b |
| 60 (%) | 81.28a | 89.03c | 97.6c | 77.6c | 12.7c | 16.30c |
| **Fertilization treatments** |  |  |  |  |  |  |
| T1 | 67.24i | 78.25i | 99.79i | 98.4i | 12.92i | 19.05i |
| T2 | 71.44h | 83.34h | 105.7h | 106.4h | 13.25h | 19.36h |
| T3 | 74.44g | 86.57g | 113.1g | 113.8g | 13.44g | 19.54g |
| T4 | 78.32f | 89.9f | 119.5f | 120.1f | 13.76f | 19.83f |
| T5 | 81.07e | 93.75e | 127.9e | 128.5e | 13.97e | 20.08e |
| T6 | 82.79d | 96.02d | 138.06d | 152.2d | 14.28d | 20.34d |
| T7 | 84.05c | 99.96c | 147.89c | 162.0c | 14.72c | 20.87c |
| T8 | 84.99b | 102.4b | 153.4b | 167.6b | 15.14b | 21.25b |
| T9 | 85.9a | 103.5a | 158.07a | 172.2a | 15.4a | 21.52a |

Abbreviations: T1 (tap water applications) (as control), T2 (soil applications of ammonium nitrate = 200 kg N ha^-1^), T3 (soil applications of ammonium nitrate = 250 kg N ha^-1^), T4 (foliar applications of potassium humate = 200 g 100 L^-1^ water), T5 (foliar applications of potassium humate = 400 g 100 L^-1^ water), T6 (soil applications of ammonium nitrate + foliar applications of potassium humate = 200 kg N ha^-1^+ foliar applications of potassium humate =200 g 100 L^-1^ water), T7 (soil applications of ammonium nitrate + foliar applications of potassium humate = 200 kg N ha^-1^+ foliar applications of potassium humate = 400 g 100 L^-1^ water), T8 (soil applications of ammonium nitrate + foliar applications of potassium humate = 250 kg N ha^-1^+ foliar applications of potassium humate = 200 g 100 L^-1^ water), and T9 (soil applications of ammonium nitrate + foliar applications of potassium humate = 250 kg N ha^-1^+ foliar applications of potassium humate = 400 g 100 L^-1^ water)

**Supplementary Table S6** The individual effects of adopting irrigation levels and fertilization (ammonium nitrate and potassium humate) treatments on (yield, and WUE) of carrots during the growing seasons of 2019/2020 and 2020/2021

| **Studied factors** | **Root yield**  **(kg ha^-1^)** | **WUE**  **(kg m^-3^)** |
| --- | --- | --- |
| **Irrigation levels** |  |  |
| 100 (%) | 23513.8b | 4.53b |
| 80 (%) | 23784.0a | 5.80a |
| 60 (%) | 13610.7c | 4.42c |
| **Fertilization treatments** |  |  |
| T1 | 8739.7h | 2.10i |
| T2 | 10405.3g | 2.51h |
| T3 | 13384.2f | 3.31g |
| T4 | 20590.8e | 4.92f |
| T5 | 23113.3d | 5.54e |
| T6 | 25548.7c | 6.13d |
| T7 | 26967.6b | 6.53b |
| T8 | 28671.3a | 6.90a |
| T9 | 25549.6c | 6.30c |

Abbreviations: WUE: water use efficiency; T1 (tap water applications) (as control), T2 (soil applications of ammonium nitrate = 200 kg N ha^-1^), T3 (soil applications of ammonium nitrate = 250 kg N ha^-1^), T4 (foliar applications of potassium humate = 200 g 100 L^-1^ water), T5 (foliar applications of potassium humate = 400 g 100 L^-1^ water), T6 (soil applications of ammonium nitrate + foliar applications of potassium humate = 200 kg N ha^-1^+ foliar applications of potassium humate =200 g 100 L^-1^ water), T7 (soil applications of ammonium nitrate + foliar applications of potassium humate = 200 kg N ha^-1^+ foliar applications of potassium humate = 400 g 100 L^-1^ water), T8 (soil applications of ammonium nitrate + foliar applications of potassium humate = 250 kg N ha^-1^+ foliar applications of potassium humate = 200 g 100 L^-1^ water), and T9 (soil applications of ammonium nitrate + foliar applications of potassium humate = 250 kg N ha^-1^+ foliar applications of potassium humate = 400 g 100 L^-1^ water).
